# A Structural Insight Into Two Important ErbB Receptors (EGFR and HER2) and Their Relevance to Non‐Small Cell Lung Cancer

**DOI:** 10.1002/ardp.202400992

**Published:** 2025-04-07

**Authors:** Edanur Topalan, Ahmet Büyükgüngör, Melih Çiğdem, Sinan Güra, Belgin Sever, Masami Otsuka, Mikako Fujita, Hasan Demirci, Halilibrahim Ciftci

**Affiliations:** ^1^ Department of Molecular Biology and Genetics Koc University Istanbul Türkiye; ^2^ Department of Molecular Biology and Genetics Istanbul Technical University Istanbul Türkiye; ^3^ Department of Biological Sciences Middle East Technical University Ankara Türkiye; ^4^ Graduate School of Biology & Health Université Paris Saclay Orsay France; ^5^ Department of Pharmaceutical Chemistry, Faculty of Pharmacy Anadolu University Eskisehir Türkiye; ^6^ Medicinal and Biological Chemistry Science Farm Joint Research Laboratory, Faculty of Life Sciences Kumamoto University Kumamoto Japan; ^7^ Department of Drug Discovery Science Farm Ltd. Kumamoto Japan; ^8^ Department of Molecular Biology and Genetics Mehmet Akif Ersoy University Burdur Türkiye; ^9^ Department of Bioengineering Sciences Izmir Katip Celebi University Izmir Türkiye

**Keywords:** epidermal growth factor receptor (EGFR), HER2, non‐small cell lung cancer (NSCLC), protein structure, tyrosine kinase domain (TKD), tyrosine kinase domain mutations, tyrosine kinase inhibitors (TKIs)

## Abstract

The epidermal growth factor receptor (EGFR) family, comprising receptor tyrosine kinases (RTK) such as EGFR and HER2, plays a critical role in various signaling pathways related to cell proliferation, differentiation, and growth. EGFR overactivation due to aberrant signaling can lead to various cancers, including non‐small cell lung cancer (NSCLC). To develop treatment for EGFR‐related NSCLC, several tyrosine kinase inhibitors (TKIs) were designed: gefitinib, erlotinib, as first‐generation; neratinib, dacomitinib as second‐generation; osimertinib, lazertinib as third‐generation, as examples. However, due to the acquired resistance by the mutations such as EGFR^T790M^ and EGFR^C797S^ together with the exon 20 insertion mutations, these drugs do not provide promising results for NSCLC patients. The development of fourth‐generation inhibitors like EAI045 and further innovative drugs to overcome this resistance problem is a must to cure EGFR‐related NSCLC. Among these, pyrazoline‐thiazole scaffolds are found effective as EGFR‐HER2 inhibitors against NSCLC, making them promising drug candidates. Although structures obtained so far for the EGFR family provide meaningful insights into the mechanisms, the quality and the quantity of the EGFR family structures are insufficient to elucidate the complete structures and functions to overcome NSCLC. This review evaluates the structures of EGFR‐HER2 and investigates their relation to NSCLC.

## Graphical abstract

This review evaluates the structures of epidermal growth factor receptor (EGFR) family members and investigates their relation to non‐small cell lung cancer (NSCLC). The development of fourth‐generation inhibitors like EAI045 and further innovative drugs to overcome this resistance problem is a must to cure EGFR‐related NSCLC. Figure created using BioRender and PyMOL.

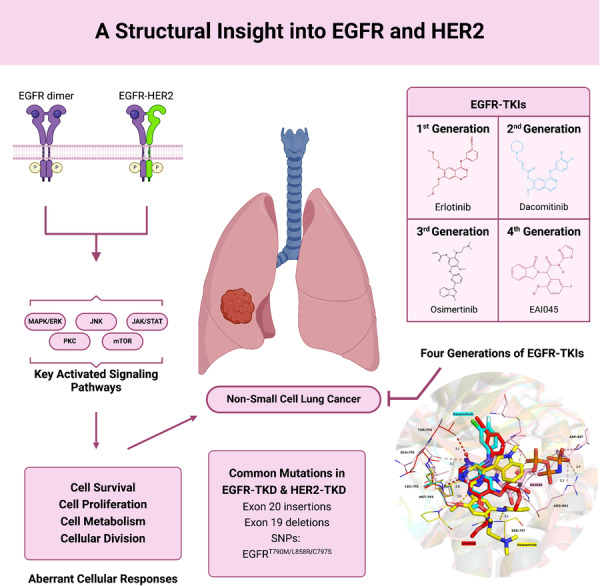

## 1 Introduction

### 1.1 Overview of the ErbB Family

Receptor tyrosine kinases (RTKs), a specialized type of tyrosine kinase, are responsible for transferring a phosphate group from ATP to the hydroxyl group of tyrosine residues. There are 58 known RTKs in humans, classified into 20 subfamilies [1]. RTKs play a vital role in cell communication and regulate complex biological processes such as cell growth, movement, differentiation, and metabolism. Abnormal RTK signaling is linked to several human diseases, particularly cancer [2].

The RTK protein family includes various subfamilies, such as epidermal growth factor receptors (ErbBs or EGFRs), vascular endothelial growth factor receptors (VEGFRs), fibroblast growth factor receptors (FGFRs), insulin receptor (IR), and insulin‐like growth factor receptor (IGFR), platelet‐derived growth factor receptors (PDGFRs), nerve growth factor receptors (NGFRs) and hepatocyte growth factor receptors (HGFRs) [3]. This review examines the structural features and clinical relevance of EGFR and HER2 within the ErbB family.

The ErbB receptor family also referred to as the EGFR family and subclass I of the RTK superfamily, comprises four members: The family comprises four members: EGFR/ErbB1/HER1, ErbB2/HER2, ErbB3/HER3, and ErbB4/HER4. These receptors share a common structural framework consisting of five main domains: an extracellular domain (ECD) responsible for ligand binding, a single transmembrane domain (TMD), an intracellular juxtamembrane domain (JMD), a tyrosine kinase domain (TKD), and a C‐terminal tail (CT) that undergoes autophosphorylation to activate various downstream signaling pathways [2]. Despite their similar structures, the receptors exhibit distinct functional characteristics. For example, HER2 lacks a known ligand and is naturally predisposed to dimerization, whereas EGFR requires specific ligand binding for activation. EGFR binds ligands such as EGF, TGF‐α, and amphiregulin (AR) to its ECD, which triggers receptor dimerization [4, 5]. HER3 binds to neuregulins 1 and 2 (NRG1, NRG2) [6], while HER4 interacts with neuregulins (NRG) 1 through 4, as well as epiregulin (EPR) and betacellulin (BTC) [7] Although HER3 has weak intrinsic kinase activity, it plays a crucial role as a coreceptor in the signaling network [8].

Ligand binding is essential for dimerization and activation of most EGFR family receptors, though HER2 uniquely remains in a ligand‐independent activation state [1]. Once ligands bind, EGFR can form either homodimers or heterodimers with other ErbB family members, including HER2. The EGFR/HER2 heterodimers are particularly significant because they show enhanced signal transduction efficiency compared with EGFR homodimers due to HER2′s ligand‐independent activation state [9, 10, 11]. Similarly, HER3 and HER4 can also dimerize with other ErbB receptors, impacting signaling pathways. This dimerization event results in the trans‐autophosphorylation of specific tyrosine residues in the CT domain, which boosts kinase activity and recruits downstream signaling proteins essential for propagating cellular responses [12, 13, 14].

**Figure 1:**
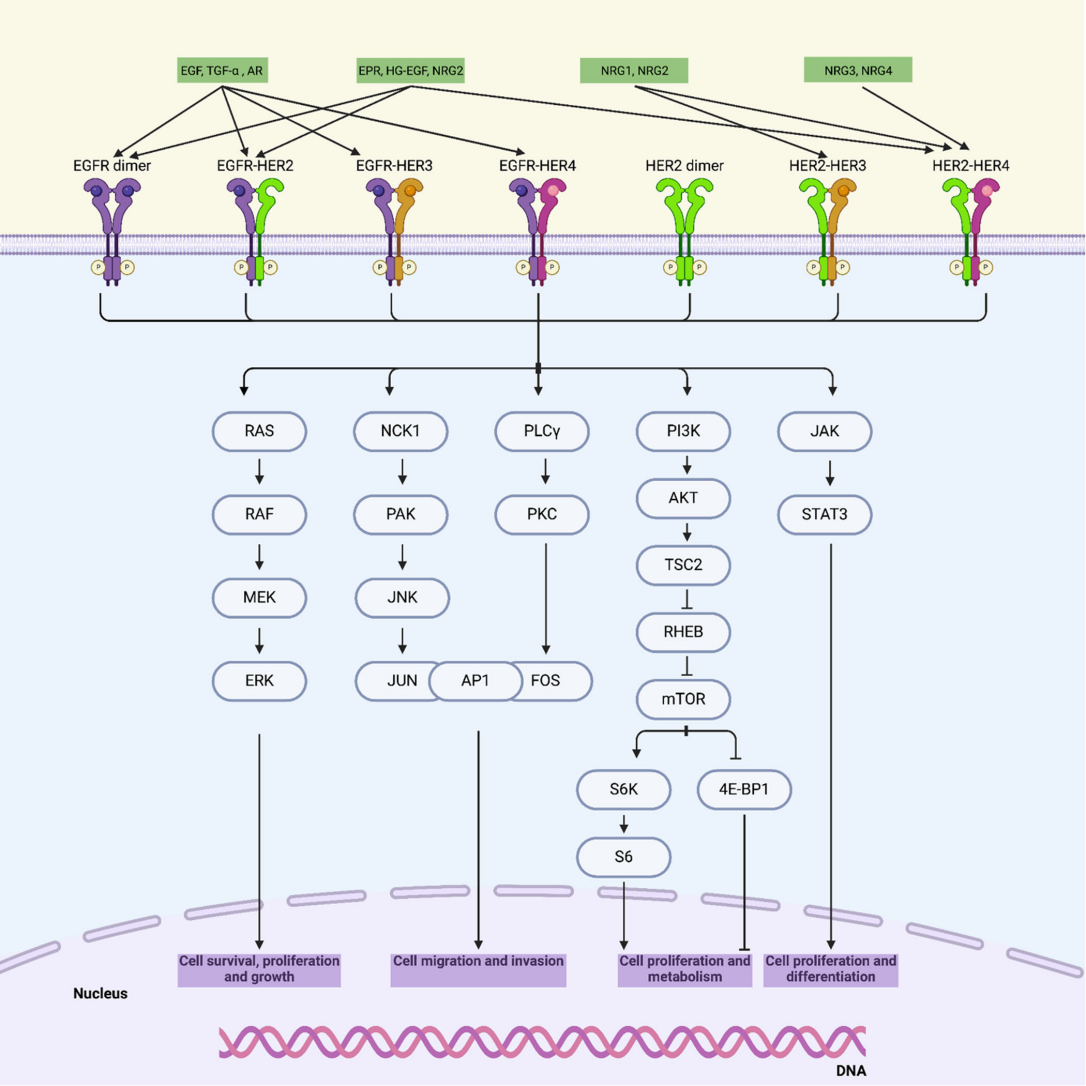
A general view of the pathway map of the ErbB family. The binding of a ligand triggers the formation of either homodimers or heterodimers among ErbB receptors, as depicted in the figure. This dimerization triggers trans‐autophosphorylation at specific tyrosine residues within the intracellular tyrosine kinase domains, denoted by circled ‘P’ symbols. This phosphorylation event initiates several downstream signaling pathways, some of which are illustrated in the figure. The Ras/Raf/MEK/ERK pathway begins with the recruitment of the adaptor protein Grb2 and the guanine nucleotide exchange factor SOS, leading to the activation of RAS, which subsequently activates RAF. RAF then phosphorylates and activates MEK, which in turn phosphorylates ERK, enabling its nuclear translocation. Once in the nucleus, ERK phosphorylates transcription factors, such as ELK1, promoting gene transcription involved in cell survival and proliferation. In the NCK1/PAK/JNK pathway, NCK1 acts as a linker between activated receptors and PAK, which activates JNK. This activation leads to the phosphorylation of transcription factors like c‐JUN, forming the AP‐1 complex that regulates genes involved in migration and invasion. Within the PLCγ/PKC pathway, PLCγ hydrolyzes PIP2 into IP3 and DAG, the latter of which activates PKC. PKC then phosphorylates transcription factors like FOS, a component of the AP‐1 complex. The PI3K/AKT/mTOR pathway is initiated by the recruitment of PI3K to the membrane, where it phosphorylates PIP2 to PIP3, leading to AKT activation. Activated AKT then phosphorylates various substrates, including TSC2, which inhibits RHEB—a negative regulator of mTOR—thereby enhancing mTOR activation. Activated mTOR promotes protein synthesis and cell growth by phosphorylating downstream effectors such as S6K1 and 4E‐BP1, driving cell proliferation and metabolism. Finally, the JAK/STAT3 pathway involves the phosphorylation of STAT3 by JAK. STAT3 then dimerizes and translocates to the nucleus, where it drives the transcription of genes involved in proliferation, survival, and differentiation. Figure was created using BioRender.

As described in Figure 1, the activation of both EGFR and HER2 leads to several downstream signaling pathways, including the PI3K/Akt/mTOR, Ras/Raf/MEK/ERK, phosphoinositide 3‐kinase (PI3K)/protein kinase B (AKT), mitogen‐activated protein kinase (MAPK), protein kinase C (PKC), and signal transducers and activators of transcription (STAT) pathways. The overactivation of these pathways results in elevated levels of cell division, proliferation, and uncontrolled increases in cell metabolism, growth, migration, and survival while reducing apoptosis [15, 16].

### 1.2 The Mutations of EGFR and HER2

Genetic alterations in the genes encoding ErbB family members have been reported to cause anomalous ErbB signaling, thus resulting in tumor development in various types of cancers, including breast and lung cancers [4, 17], and those mutations allow ligand‐independent activation of EGFR which is common in non‐small cell lung cancer (NSCLC) [18].

A considerable number of lung and breast cancer cases arise from EGFR mutations, especially mutations in the EGFR‐TKD [19]. The tyrosine kinase domain of the EGFR protein is encoded by exons 18–24 within the gene [20]. Therefore, mutations affecting the EGFR‐TKD are seen within these exons, and most are responsive to EGFR‐tyrosine kinase inhibitors (EGFR‐TKIs) in patients with NSCLC. However, not all EGFR‐positive NSCLC cases can be treated with EGFR‐TKIs. For instance, exon 20 insertion mutants of EGFR are not sensitive to commonly used tyrosine kinase‐targeting drugs [21]. A volume of 85% of the mutations are either exon 21 L858R substitutions or deletions in exon 19. The remaining 15% of the mutations are the “uncommon” mutations of the EGFR‐TKD [19]. The most frequently reported “uncommon” EGFR mutations are G719X, L861X, S768I, and exon 20 insertion mutations [19].

HER2 is a critical player in cancer biology, with its role being particularly notable in breast cancer and NSCLC. In breast cancer, HER2 amplification acts as a primary driver, whereas in NSCLC, tumor progression can result from diverse mechanisms, including gene amplification, gene mutation, and HER2 overexpression [22]. Under normal conditions, active HER2 and its inactive, dephosphorylated form are in equilibrium, a balance essential for regulating cell growth, survival, and normal breast tissue development [23]. However, when HER2 becomes overactivated, this equilibrium is disrupted, leading to abnormal levels of cell survival and proliferation.

HER2 has a distinguished heterodimerization capability, which makes it crucial for downstream signal transductions and makes HER2 deregulation a critical factor in tumorigenesis [22]. HER2 overexpression and HER2 amplification are the leading alterations in NSCLC patients [24], whereas around 4% of NSCLC patients have HER2 mutations [25]. In general, HER2 overexpression occurs simultaneously with HER2 amplifications, however, in the case of NSCLC, no significant correlation was observed, and this situation leads to poor prognosis for NSCLC patients with HER2 alterations. Recent studies include HER2‐TKIs, anti‐HER2 antibodies, and antibody‐drug conjugates (ADCs), and the results are promising for HER2‐targeted NSCLC treatments [15]. HER2 mutations also have a high prevalence in the majority of breast cancer cases and are also targets of tyrosine kinase inhibitors during the treatments [26].

### 1.3 Treatment Options for NSCLC

As a disease in which EGFR mutations have a profound impact, NSCLC is a histological type of lung cancer [27], and it is responsible for around 85% of new lung cancer diagnoses [28, 29]. As the most commonly seen form of lung cancer, NSCLC contains pathological forms like adenocarcinoma, squamous cell carcinoma, and so on, and since these symptoms are generally not detectable at an early stage, approximately 37% of the patients are diagnosed at an advanced stage of the disease, a stage that has < 6% rate of 5‐year survival rate [30]. A reason why NSCLC diagnosis is challenging is the differences among patients on the alterations of the mutations. Western and Asian populations differ especially. For example, in the case of non‐squamous NSCLC, EGFR mutations are more commonly observed in Asia than in the United States and Europe [31, 32]. Genetic factors, smoking, and air pollution are some of the factors that give rise to lung cancer progression, and for the treatment of lung cancer, surgery, chemotherapy, and radiation therapy were performed for years as the traditional options [33, 34].

Surgery is a treatment option for cancer by removing the tumor; however, there is a high risk of recurrence due to the micrometastases presence. Despite advancements in prognostic tools that increase the likelihood of complete tumor resection, recurrences remain almost inevitable [35]. For the cases in which surgery is not possible for NSCLC treatment, radiotherapy is an alternative option; however, the results are inferior to those of surgery [36]. Another treatment option is chemotherapy, which is beneficial for NSCLC treatment initially [37], yet drug resistance immediately occurs after the treatment [38]. As a new approach, immunotherapy is proposed for NSCLC treatment, and studies revealed that immunotherapy has better results than chemotherapy. However, while immunotherapy has improved survival rates for patients with NSCLC, it is not always an effective solution, and there are still numerous cases where this treatment option falls short [39, 40, 41]. Moreover, immunotherapy is not helpful for the treatment of approximately 70% of patients with advanced NSCLC [42], in fact, in the case of EGFR‐mutated NSCLC, immunotherapy is not beneficial [43].

Monoclonal antibodies (mAbs) are another option for the treatment of NSCLC. EGFR‐targeted mAbs provide clinical success by preventing ligand binding of EGFR, which reduces the downstream ERK signaling [44]. However, some studies show that HER2‐targeted mAbs and conventional pan‐HER‐TKIs give unsuccessful results for the tumor treatment for NSCLC with HER2 alterations. Nevertheless, new HER2‐TKIs have more promising results than the older ones [45]. Still, the emergence of resistance by upregulation of downstream signaling and antiapoptotic pathways, and alternative RTK pathways signaling is valid for both TKIs and mAbs [44].

### 1.4 Tyrosine Kinase Inhibitors (TKIs)

Nowadays, to develop novel treatments, molecular targets are used for targeted therapy, which may be useful for specific mutations of EGFR [46], and EGFR‐TKIs started to be used for NSCLC treatment [47]. The standard treatment for NSCLC with EGFR mutations includes EGFR‐TKIs such as gefitinib, osimertinib, and afatinib [48]. Three generations of inhibitors for EGFR have been developed after the relationship between EGFR and NSCLC was discovered [49]. The drugs that are used in this treatment option basically bind to EGFR‐TKD and inhibit it explicitly [50]. EGFR‐TKIs trigger cell death by hindering downstream signaling with binding reversibly or irreversibly [11]. For instance, inhibitor drugs like gefitinib are aimed to block EGFR activation by competing with ATP to bind to the ATP‐binding domains [51]. Clinical data show that this type of NSCLC treatment is superior to traditional platinum‐based chemotherapy, however, this superiority is restricted by the resistance that emerged after the treatment [48]. Mutant versions like EGFRT790M are conformationally changed in the kinase region, therefore TKIs cannot efficiently bind to the active site of the tyrosine kinase due to steric hindrance [52]. Moreover, some traditional inhibitor drugs cannot bind to EGFR when it is in the active state, which makes them useless in cases where mutations lead to constitutive activation [53].

**Figure 2:**
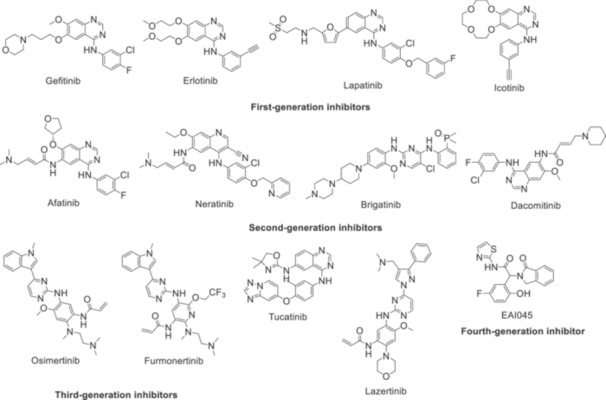
The chemical structures of first, second, third, and fourth‐generation EGFR inhibitors.

As tyrosine kinase inhibitors for the patients with NSCLC harboring EGFR mutations, gefitinib, erlotinib, lapatinib, and icotinib as first‐generation inhibitors; afatinib, neratinib, brigatinib, and dacomitinib as second‐generation inhibitors; osimertinib, furmonertinib, tucatinib, and lazertinib as third‐generation inhibitors are examples for approved drugs (Figure 2). Meanwhile, fourth‐generation inhibitors such as EAI045 (Figure 2), BLU‐701, BBT‐176 are some of the recent attempts to overcome EGFR^C797S^ and EGFR^T790M^ mutations [11, 18, 47]. The efforts to overcome the acquired resistance problem by designing novel TKIs still appears crucial to overcome EGFR‐related NSCLC.

## 2 Structural Insight Into EGFR and HER2

The high‐resolution structures of EGFR and HER2 provide insights into their functional mechanisms. Although a complete structure for full‐length ErbBs is not available yet, significant progress has been made by examining receptor fragments and reconstructing models based on these data [54]. Nevertheless, crystal structures obtained so far provide valuable information about the structures and functions of EGFR and HER2.

**Figure 3:**
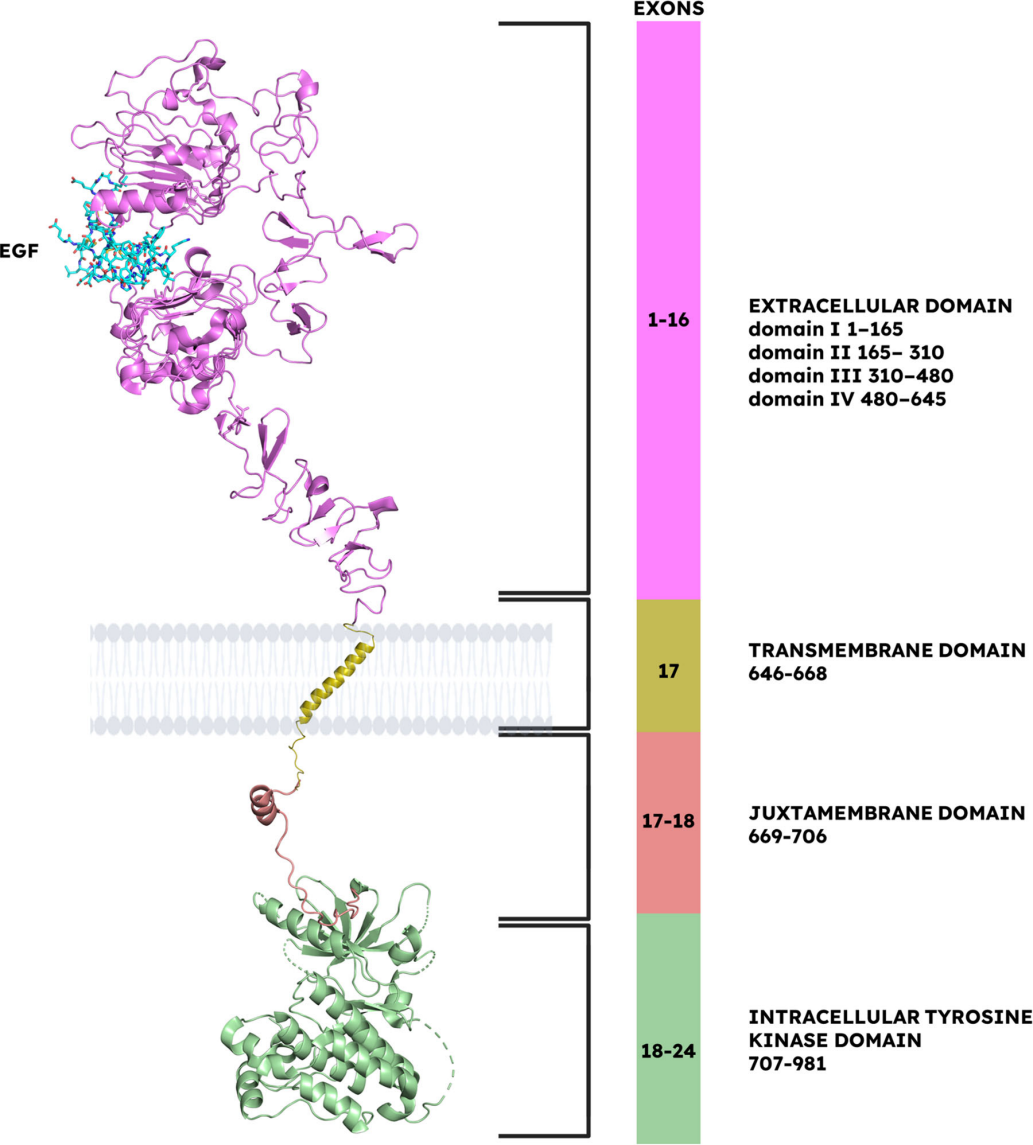
An overview of the EGFR structure. The figure illustrates a schematic representation of the epidermal growth factor receptor (EGFR) structure. The diagram highlights the extracellular domain (ECD, PDB: 3NJP), transmembrane domain (TMD, PDB: 2KS1), juxtamembrane domain (JMD, PDB: 3GOP), and intracellular tyrosine kinase domain (TKD, PDB: 3GOP). Figure created using PyMOL (PyMOL Molecular Graphics System, Version 3.0, Schrödinger LLC).

The domains that form EGFR are a ligand‐binding ECD, a short hydrophobic TMD, a juxtamembrane domain, and an intracellular tyrosine kinase domain (Figure 3) [55]. Among these, the ECD has four distinct subdomains. Domain I and domain III are leucine‐rich and share significant sequence homology, while domain II and domain IV are rich in cysteine residues. Domain II contains the dimerization arm (DA), which is pivotal for receptor activation [56]. On the other hand, domain IV features two different kinds of disulfide‐bonded modules: C1, where a bow‐shaped loop is constrained by a single disulfide bond, and C2, where four consecutive cysteine residues are connected via two disulfide bonds (C1–C3 and C2–C4), which causes the formation of a structure like a knot [4, 57]. In addition, by disrupting the intramolecular tether among the domain II and IV, a significant conformational rearrangement is induced by the EGFR ligand binding. This disruption releases the DA, enabling it to interact with domain II of another ligand‐bound receptor, thereby facilitating dimerization and the following activation. However, in the cases without the presence of a ligand, the DA remains sequestered within domain IV, maintaining the receptor in an autoinhibited conformation.

Following the ECD, the sequence of the structure is as follows: a TMD around 23 amino acid residues length, followed by a relatively short juxtamembrane domain that is mainly responsible for the tyrosine kinase domain separation from the plasma membrane. Then, the carboxy‐terminal, largely an unstructured tail (between the amino acid residues 953 and 1186), which has at least five tyrosine autophosphorylation sites, is after the tyrosine kinase domain [58].

The EGFR‐TKD includes an N‐lobe with an αC‐helix in the residues between 729 and 744, and five β‐strands (β1‐5). This lobe is paired with a C‐lobe that contains five different α‐helices (αE, αF, αG, αH, αI) [59]. Among these lobes, under a conserved loop that is rich in glycine, connects the β1‐β2 links in the N‐lobe, and is involved in phosphate binding, an ATP‐binding site is established in a cleft. Through the backbone, the interactions between this loop and ATP phosphatases occur [60].

When it is activated, an ionic bond is formed between Glu738 within the αC helix and Lys721 in the β3 strand, which leads to ATP phosphate group interactions. The C‐lobe encircles the base of the ATP‐binding cleft and stabilizes the conserved catalytic loop between the residues Asp812 and Asn818. Within this loop, the interactions between the tyrosine substrate's hydroxyl group and Asp812 facilitate its nucleophilic attack, while Asn818 stabilizes Asp812 through hydrogen bond formation [61]. The C‐lobe also plays a role in the regulatory activation loop between the residues Asp831 and Val852, which features an Asp‐Phe‐Gly (DFG) motif at its base [62].

By inhibiting the kinase activity when the autophosphorylation does not take place, the C‐terminal domain of EGFR, comprising the residues between 982 and 1210, is critical for receptor activation regulation [63, 64]. Another property of this domain is that it contains phosphorylation sites in the residues which are rich in proline [55]. The flexibility of this domain's structure results in disorder within EGFR crystal structures that are published, particularly in the polypeptide chain (between the residues 990 and 1005), which shows significant fluctuations in molecular dynamics simulations [64].

### 2.1 Overview of the Structures of EGFR Homodimer and EGFR‐HER2 Heterodimer

As previously mentioned, ECD dimerization is crucial for activating ErbB receptors, leading to intracellular signaling. EGFR can form both homo‐ or heterodimers. Interestingly, in normal conditions where EGFR and HER2 are more or less expressed in the same quantity, EGF readily induces homodimerization but not EGFR‐HER2 heterodimerization, which indicates that homodimerization is more favored. ECD is sufficient for EGF‐dependent EGFR homodimerization, but not EGFR‐HER2 heterodimer [65, 66].

**Figure 4:**
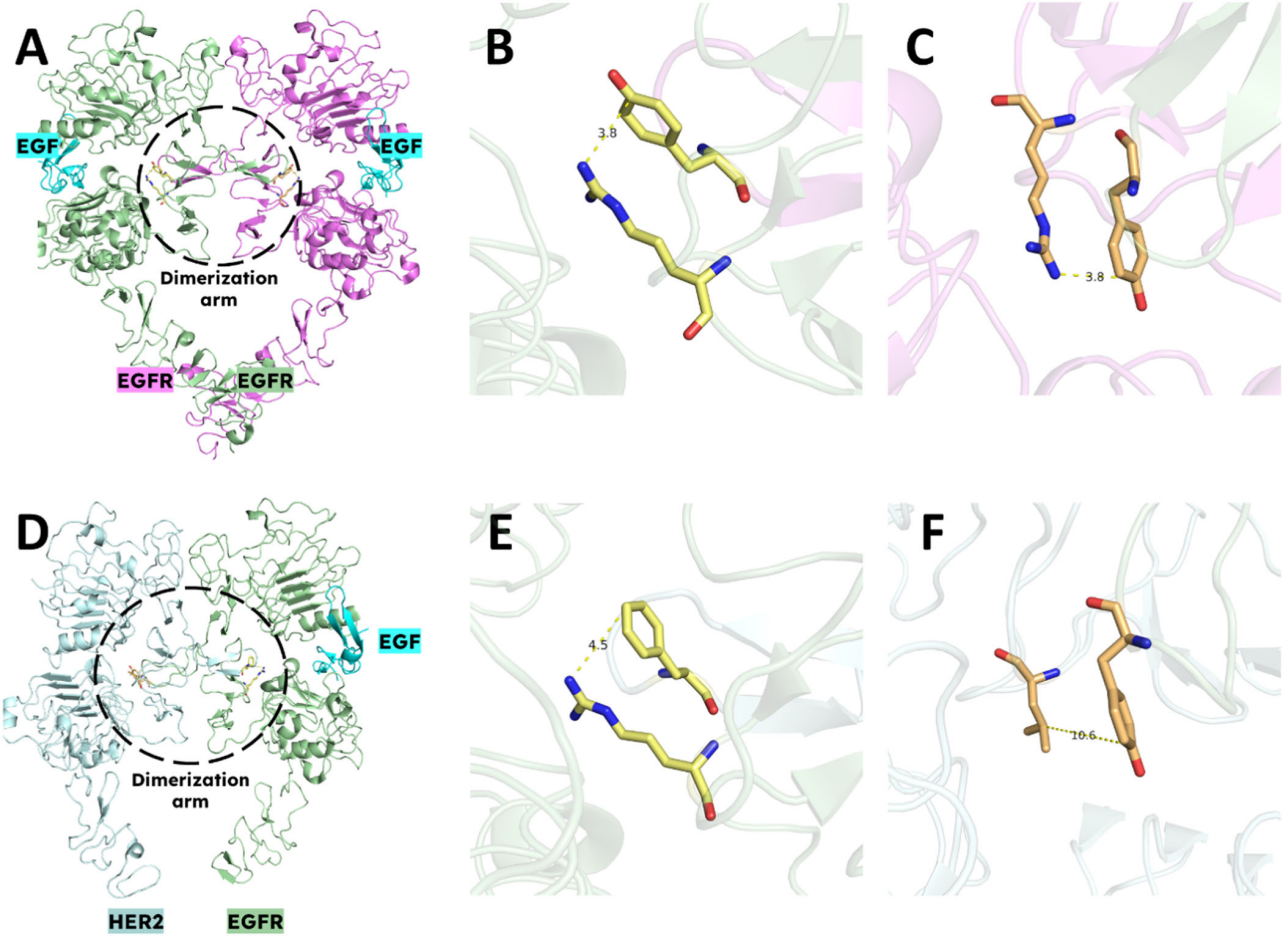
Ectodomain structures of EGFR homodimer (A, B, C) and EGFR–HER2 heterodimer (D, E, F). (A) A general view of the complete EGFR homodimer, with one EGF bound to each one of EGFRs. One EGFR ectodomain is shown in palegreen, and the other one in violet. EGFs are shown in cyan. The dimerization arms (DA) are highlighted with a black dashed circle. (B) A zoom into one of the interactions between the DA of one EGFR and the binding pocket (BP) of the other EGFR. The Y250 from the DA of the violet EGFR and the R284 from the BP of the green EGFR form a *π*–cation interaction, stabilizing the dimer and thus favoring the dimerization. (C) Symmetrical figure to (B), with the Y250 of one EGFR and R284 of the other EGFR forming a *π*–cation interaction, thus allowing for dimerization. (D) A general view of the complete EGFR‐HER2 heterodimer, with EGF bound to EGFR. The EGFR ectodomain is shown in palegreen, and HER2 in palecyan. EGF is shown in cyan. The DAs are highlighted with a black dashed circle. (E) A zoom into the interactions between the DA of HER2 and the BP of EGFR. The F278 from the DA of HER2 and the R308 of the BP of EGFR form a *π*–cation interaction, stabilizing the dimer and thus favoring the dimerization. This interaction is crucial for heterodimerization. (F) A zoom into the DA of EGFR inserted into the BP of HER2. The Y274 of the DA of EGFR cannot stabilize the heterodimer since the L312 of the BP of HER2 (which substitutes the R284 of EGFR in the HER2 structure) does not possess a *π* system to interact with the Y274, thus the residues are too far one from another and the dimer is not stabilized by these residues. Figure created using PyMOL (PyMOL Molecular Graphics System, Version 3.0, Schrödinger LLC).

Dimerizations are energetically favorable when there are key noncovalent interactions (like hydrogen bonds, Van der Waals forces, *π*‐stackings, and *π*‐cation interactions) are present between residues from the DA of one monomer and residues from the binding pocket (BP) of another monomer. Those residues should be well‐placed to ensure the dimerization phenomenon. The EGFR homodimer is symmetrical, and both EGFR monomers participate equally in the dimerization. The Tyr250 (Y250) from one EGFR monomer forms a *π*–cation interaction with the Arg284 (R284) with another one as seen in Figure 4. They are at 3.8 Å from one another, which is an ideal distance for forming a *π*–cation interaction between the aromatic ring of tyrosine and positively charged arginine. This interaction happens in each of the two DAs and is responsible for the homodimerization. On the other hand, the EGFR‐HER2 heterodimer is asymmetrical. In HER2, the Tyr250 and Arg284 become Phe278 and Leu312, respectively. The F278 in the HER2 DA is still capable of forming a *π*–cation interaction with R308 of EGFR BP. Nevertheless, the Y274 of EGFR DA no longer has a *π*–cation interaction partner, which results in the weakening of the dimer. This explains why homodimers are generally more favorable than heterodimers, as the stronger, symmetrical interactions in homodimers like EGFR‐EGFR lead to greater stability [67].

Interestingly, although the major mediator in HER dimer formation is domain II of the ECD of HER family members, distinct interaction patterns are observed for symmetrical (homodimers) and asymmetrical dimers (heterodimers) [68]. Symmetric dimers depend primarily on DA‐mediated contacts, whereas asymmetric dimers engage more extensively with the N‐terminal region. In the EGFR/HER2 complex, both the N‐ and C‐terminal regions of Domain II exhibit significantly larger buried surface areas (BSA) compared with the EGFR homodimer, indicating a more intimate interaction. As illustrated in Figure 3, HER2's DA has a much better fit and locks in, whereas EGFR's DA shows minimal contact and, as a consequence, greater flexibility. Thus, in the EGFR HER2 heterodimer, the well‐placed HER2 DA is crucial for dimer stabilization, while the more flexible EGFR DA appears less significant, reflecting the general pattern observed across different HER family dimers where the unbent subunit's DA plays a key role, and the bent subunit's DA varies in its interactions [67]. The role of EGFR–HER2 heterodimer in NSCLC is nowadays even more apparent, as the signaling pathways are remarkably enhanced and prolonged by heterodimers compared with homodimers [69, 70]. HER2, unlike EGFR, does not alter its membrane dynamics or undergo endocytosis since it is less prone to the internalization process [71, 72]. Instead, it assists in keeping EGFR on the plasma membrane. Consequently, HER2 likely prevents the rapid endocytosis and degradation of EGFR following activation, extending downstream phosphorylation signals that encourage cell growth and proliferation, ultimately leading to tumorigenesis [67].

The phenomenon of ECD dimerization is certainly important for EGFR activation since it constitutes the first step to tyrosine kinase domain activation. The activation of the TKD is what will allow the trans‐phosphorylation and the signaling cascade, which in turn may result in tumorigenesis.

### 2.2 Conformational Dynamics of Active and Inactive EGFR Tyrosine Kinase Domains

**Figure 5:**
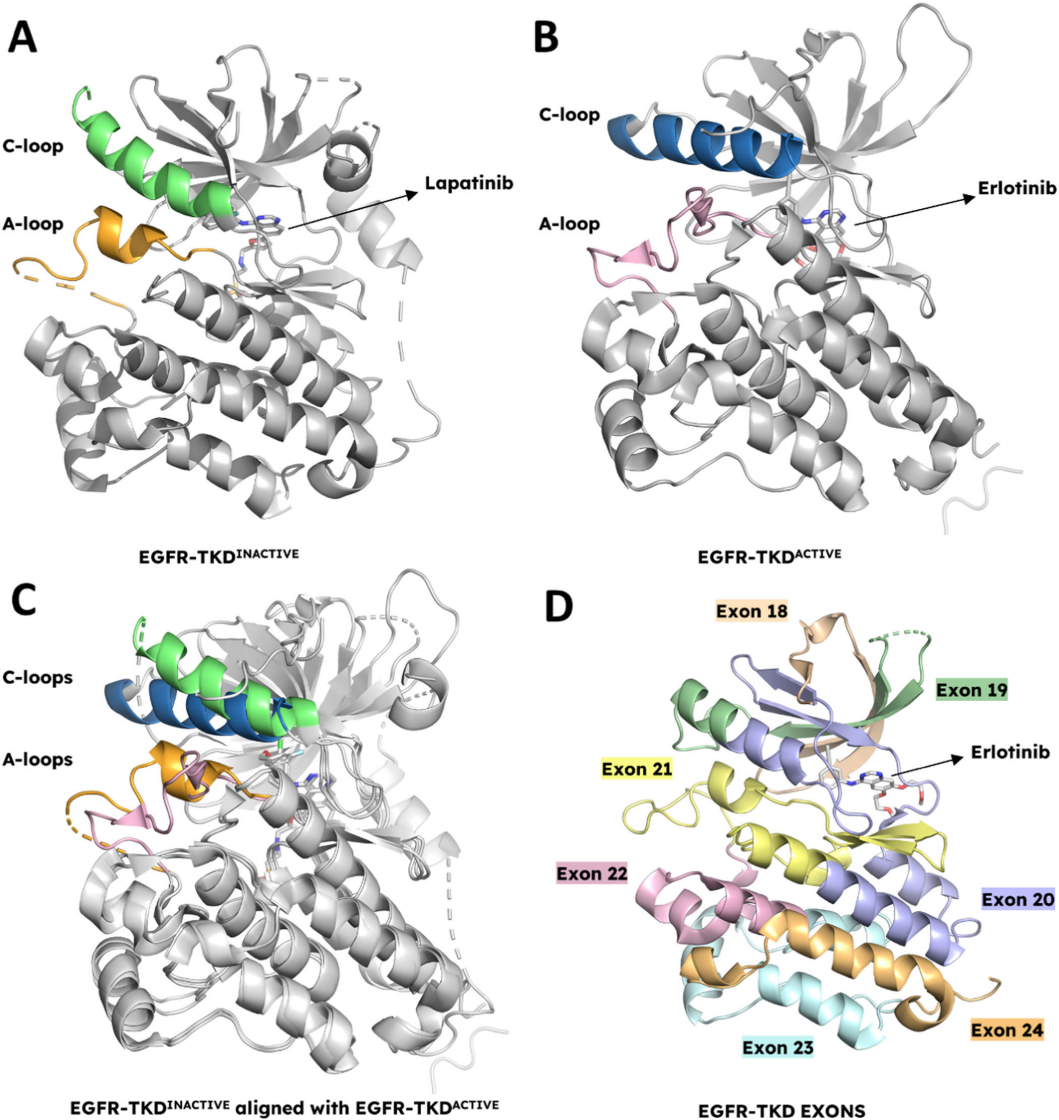
EGFR tyrosine kinase domain structures. (A) Inactive conformation of EGFR tyrosine kinase domain (EGFR‐TKD^Lapatinib^, PDB: 1XKK) includes a small helical turn within the activation loop (orange) and an outward rotated helix αC (green). (B) Active conformation of EGFR tyrosine kinase domain (EGFR‐TKD^Erlotinib^, PDB: 1M17) contains a unique extended activation loop (orange) and features an inwardly rotated helix αC (green). (C) Alignment of inactive and active conformations of EGFR tyrosine kinase domains. (D) EGFR tyrosine kinase domain exon map. EGFR exon 18 in wheat, exon 19 in green, exon 20 in blue, exon 21 in yellow, exon 22 in pink, exon 23 in cyan, and exon 24 in orange (EGFR‐TKD^ERLOTINIB^, PDB: 4HJO). Figure created using PyMOL (PyMOL Molecular Graphics System, Version 3.0, Schrödinger LLC).

Understanding the inactive (Figure 5a) and active (Figure 5b) conformational states of EGFR‐TKD is essential for designing new inhibitors. The transition between these states is a critical process that regulates receptor activation and has significant implications for developing therapeutic strategies for cancers with dysregulated EGFR activity. The EGFR‐TKD alternates between inactive and active conformations and is regulated by ligand binding [54, 73], dimerization [74], internalization [75], and degradation [76, 77]. Ligand binding triggers receptor dimerization, leading to the formation of an asymmetric dimer of the intracellular kinase units. The formation of an asymmetric kinase domain dimer is crucial for its function. The early crystal structures of EGFR‐TKD showed a conformation similar to that of an active kinase [59], marked by the specific positioning of the activation loop and the C‐helix in the N‐terminal lobe. This active conformation is consistent with EGFR's distinct characteristic of functioning without the need for activation‐loop phosphorylation [78].

The human genome encodes over 500 protein kinases, each featuring a highly conserved catalytic domain with α‐helix C‐terminal and β‐strand N‐terminal lobes that function as ATP‐binding sites [79]. The activation loop of these kinases includes residues such as tyrosine, threonine, or serine that undergo phosphorylation to regulate kinase activity [80]. Two key regions are typically used to determine whether a kinase is in an active or inactive state: the αC helix and the DFG motif within the activation segment [61]. In the active conformation of EGFR, the kinase domain, and the αC helix, spanning residues 753–767, are twisted inward towards the N‐lobe and the active site. This orientation reduces the distance between Glu762 on the αC helix and Lys745 on the β3 strand, facilitating the formation of a salt bridge and enhancing interactions with the α‐ and β‐phosphate groups of ATP [81].

In summary, the transition from the inactive to the active conformation in EGFR‐TKD involves outward rotation of the A‐loop and inward rotation of the C‐loop. These structural changes are crucial for regulating the kinase's activity and are essential for understanding its role in signal transduction and its implications for targeted therapies.

### 2.3 Structural Insight to the Most Commonly Seen EGFR Tyrosine Kinase Domain Mutations

**Figure 6:**
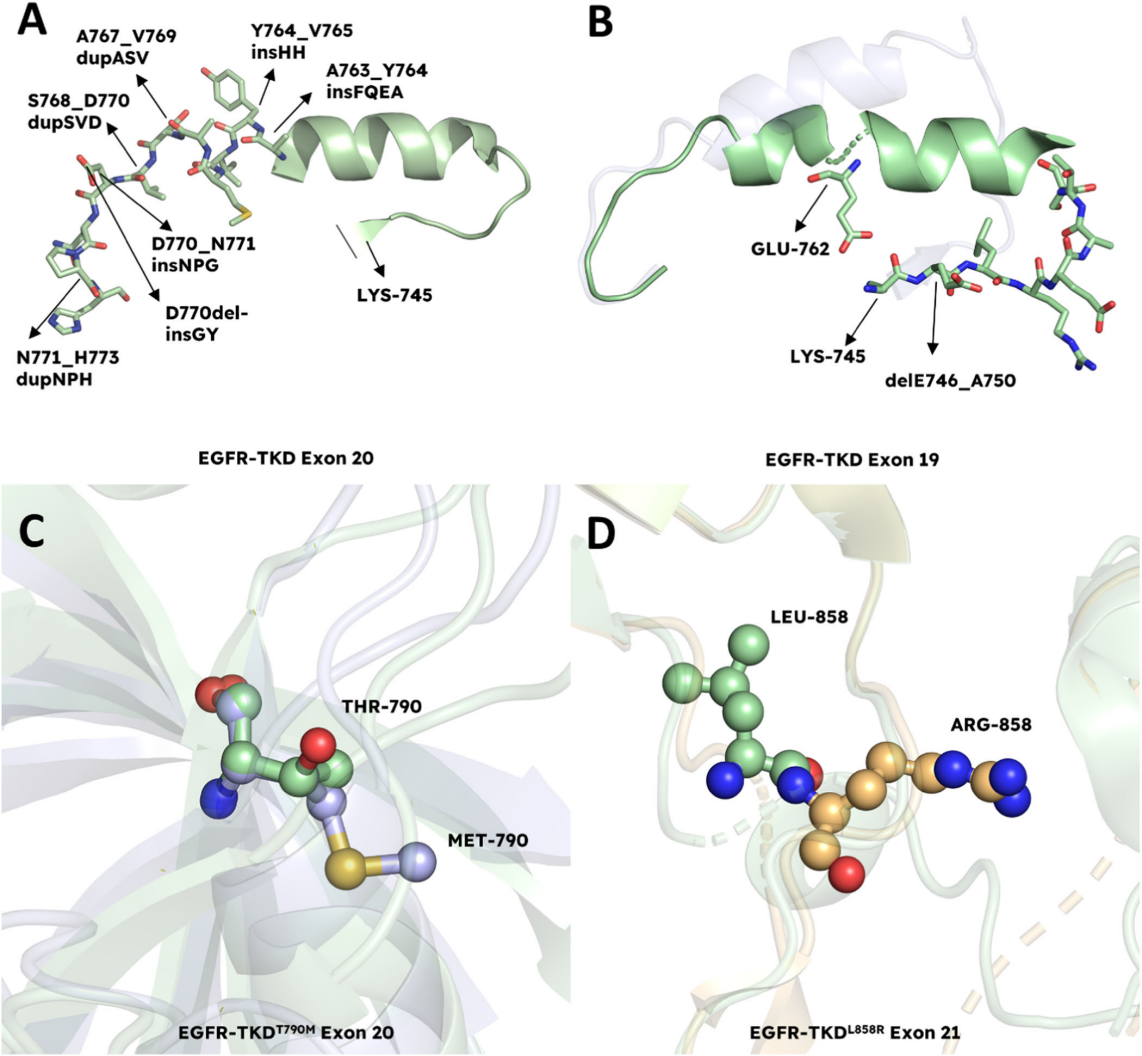
EGFR tyrosine kinase domain mutations. (A) Most commonly seen EGFR insertion mutations in exon 20 (PDB: 1M17). (B) ELREA motif deletion mutations of EGFR in exon 19. These mutations maintain the kinase in its active conformation (palegreen, PDB: 1M17) by limiting the flexibility of helix αC, which is essential for adopting the inactive state (faded, PDB: 2JIV). Deletion mutation between the residues E746 and A750 leads to the formation of interaction between the residues L745 and G762, which restricts the flexibility. (C) EGFR^WT^ (PDB: 2J6M) in palegreen, EGFR^T790M^ (PDB: 2JIT) in lightblue. The T790M mutation of EGFR is the most common mechanism of acquired resistance against inhibitors. (D) EGFR^WT^ (PDB: 4HJO) in palegreen, EGFR^L858R^ (PDB: 6JWL) in light orange. L858R mutation, a common mutation seen in exon 21 of EGFR. Figure created using PyMOL (PyMOL Molecular Graphics System, Version 3.0, Schrödinger LLC).

Exon 20 insertion mutations of EGFR contain diverse insertions ranging from 1 to 7 amino acids that emerge near the C‐terminal end of the αC‐helix (Figure 6a) [82]. These insertion mutations are seen in NSCLC very frequently, making up 4%–10% of all recorded EGFR mutations [53, 83]. It has been revealed by the crystal structures of exon 20 insertion mutants of EGFR that they develop a wedge that prevents the αC‐helix from rotating outward and conforming into inactive form; hence, resulting in constitutive activation of kinase domain [84]. Additionally, it has been found that exon 20 insertion mutations differ in terms of their responsiveness against EGFR‐TKIs; for example, A763_764insFQEA has a higher affinity than D770_N771insNPG for the first‐generation EGFR‐TKIs [82].

Exon 19 deletion mutations are another type of mutation that is responsive to EGFR‐TKIs [85]. These mutations are the most prevalent ones similar to L858R (seen in 40% of the cases) and are observed in around 50% of the patients [86]. ELREA deletion mutation in exon 19 (delE746_A750) (Figure 6b) is frequently seen in NSCLC patients [87]. The essential role of ELREA is to improve the flexibility of the regulatory region helix αC for switching between active and inactive states of the kinase domain. Modeling studies imply that ELREA deletion results in reduced helicity in the N‐terminal portion of the helix, leading to the formation of a firm β3 − αC loop [88]. This brings about a distinct Glu762‐Lys745 salt bridge and hence, it is understood that alteration to helix αC prevents transition to the inactive state, keeping the kinase domain in active form [89].

As previously mentioned, the T790M gatekeeper mutation is the most frequent mechanism of acquired resistance to first‐generation EGFR‐TKIs. (Figure 6c) [90]. The gatekeeper mutation results in activation of the signaling pathway by elevating the ATP affinity and spatially impeding drug binding, resulting in resistance to first‐generation inhibitors [91]. Moreover, T790M mutants do not require receptor dimerization for constitutive activation of the pathways [92, 93, 94, 95].

The majority of the EGFR mutations are either activating L858R (Figure 6d) or exon 19 deletion mutations (Figure 5b); nevertheless, the uncommon mutations still occur in the gene encoding exons 18‐25 of EGFR in NSCLC and are not as sensitive as above‐mentioned mutations to EGFR‐TKIs [96]. EGFR protein with L858R and exon 19 deletion mutations have increased sensitivity for TKIs, which allows them to outcompete ATP molecules, therefore blocking receptor activation [97].

### 2.4 Structural Analysis of HER2, Its Activation Mechanisms, and Common HER2 Mutations In NSCLC

HER2 is mainly activated through an allosteric mechanism, where the dimerization of its kinase domain and that of its dimerization partner stabilizes the HER2 tyrosine kinase domain (HER2‐TKD) in its active conformation. Unraveling the structure of HER2‐TKD enlightens the molecular mechanisms related to the activation processes and the options for selective inhibition. The N‐terminal lobe of HER2‐TKD primarily consists of β‐strands and a single α‐helix, while the C‐terminal lobe of HER2‐TKD is mainly α‐helical. The link between these lobes occurs due to a hinge region which is flexible, whereas a deep cleft that forms an ATP‐binding site separates these lobes. The positioning of these lobes affects the size of the ATP‐binding site, with variations dependent on the activation state of the HER2‐TKD. The residues involved in catalytic activity are mostly situated near the cleft such as the α‐helix C (Pro761‐Ala775), and the Gly‐rich nucleotide phosphate‐binding loop (Leu726‐Val734) in the N‐terminal lobe; and the activation loop (Asp863‐Val884), the catalytic loop (Arg844‐Asn850), and the DFG motif (Asp863‐Gly865) in the C‐terminal lobe [98].

**Figure 7:**
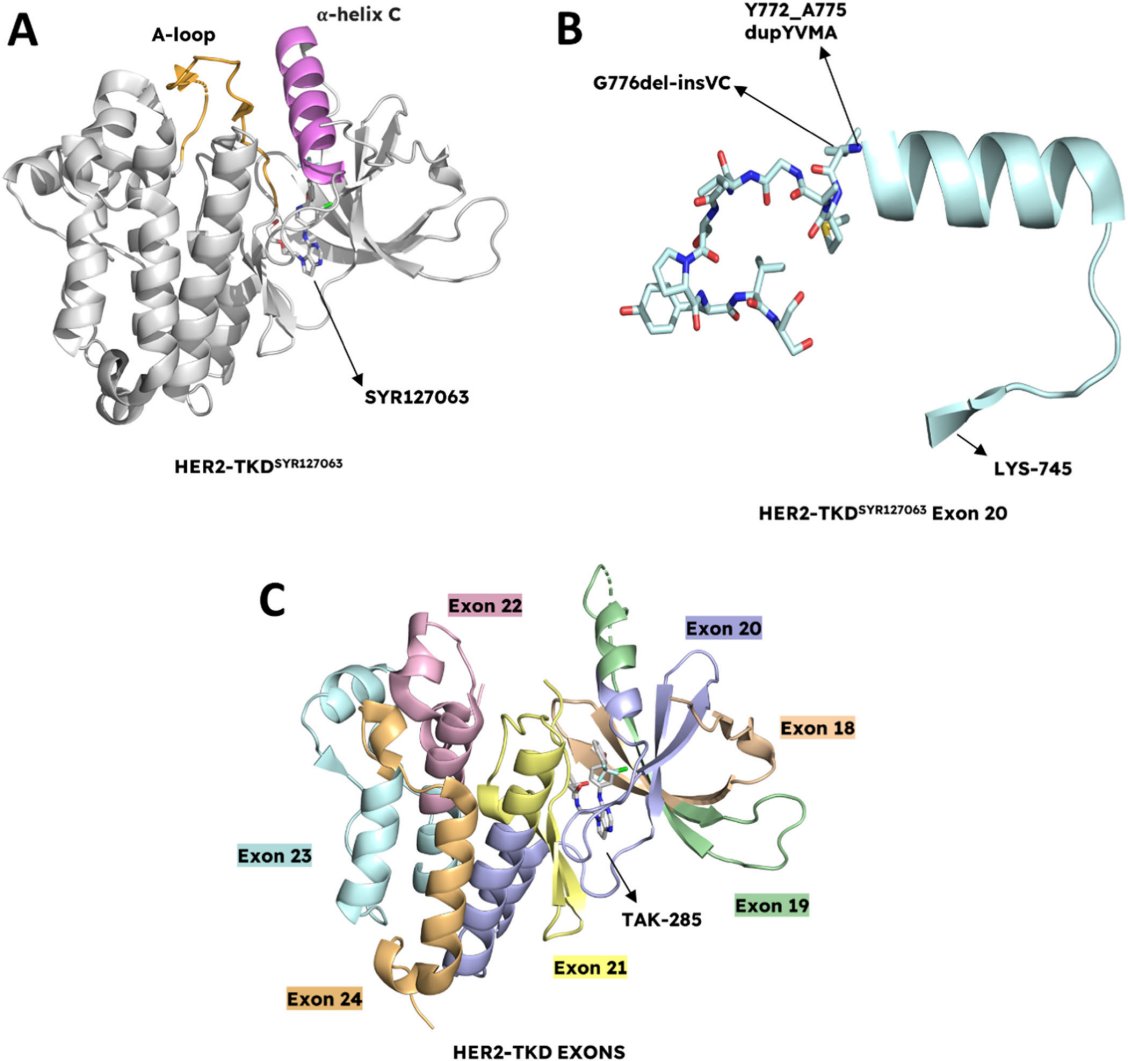
HER2 tyrosine kinase domain structure. (A) HER2 tyrosine kinase domain (HER2‐TKD^SYR127063^, PDB: 3PP0) contains an alpha‐helix C (pink) and A‐loop (orange). HER2 is in active‐like conformation with extended A‐loop and alpha‐helix C which contains the residues related to catalytic activity. (B) Most commonly seen HER2 insertion mutations in exon 20 (PDB: 3PP0). (C) HER2 tyrosine kinase domain exon map. HER2 exon 18 in wheat, exon 19 in green, exon 20 in blue, exon 21 in yellow, exon 22 in pink, exon 23 in cyan, and exon 24 in orange (HER2‐TKD^TAK‐285^, PDB: 3RCD). Figure created using PyMOL (PyMOL Molecular Graphics System, Version 3.0, Schrödinger LLC).

Principally, in the active state of HER2, the kinase domain is in a conformation in which the α‐helix C is orderly positioned, and the activation loop is extended without forming any secondary structures, which allows the catalytic activity, even in the absence of phosphorylation. On the other hand, in the inactive state of HER2, the α‐helix C is displaced away from the active site, and the activation loop acquires a more structured conformation, which prevents catalytic activity. However, unlike the other ErbB family members, the obtained crystal structures for HER2 do not show a clear distinction between active and inactive states. Therefore, HER2 structures are referred to as “intermediate active‐inactive conformation” or “active‐like conformation.” Mainly, the orientation of the α‐helix C and the extended conformation of the activation loop in the HER2‐TKD result in this situation **(**Figure 7a). The flexibility of the α‐helix C leads HER2 to adopt several conformations, including inactive states. This enhanced the flexibility of the HER2‐TKD active site conformation depends on its unique Gly‐rich region after the α helix C‐β4 loop, which is related to HER2's low intrinsic catalytic activity compared to the other members of the ErbB family. In the case of active EGFR conformation, H‐bond interactions, which are essential for catalysis by stabilizing the orientation of the α‐helix, are formed by Ser768 and Asp770 in this loop, however, these interactions are hindered by the Gly‐rich region in HER2 [98]. Moreover, the HER2‐TKD activation loop is well‐ordered, and it does not form a secondary structure in this active‐like conformation [99, 100], which is a significant feature of its active conformation. On the other hand, another crucial activation mechanism is the formation of salt bridges between the residues Lys753 in β‐strand 3 and Glu770 in α‐helix C. Active and inactive HER2 states distinguish on the distances of the salt bridges, and in the available structures of HER2‐ TKD, these distances were found as 6.7 Å (HER2‐TKD^SYR127063^, PDB: 3PP0) and 10.1 Å (HER2‐TKD^TAK‐285^, PDB: 3RCD) indicating an intermediate conformation among the active conformation and the inactive conformation [101]. For instance, the disruption of salt bridges causes the α‐helix C to shift 20° away from the kinase active site in the HER2‐TKD^SYR127063^ structure [98].

HER2 mutations are generally observed in the tyrosine kinase domain, and they are the leading factors in various conformational changes in the ATP‐BP that increase kinase activity and several oncogenic pathways [102]. Most of the mutations are insertions and deletions between Tyr772 and Pro780 however, some point mutations and other insertions have also been observed, and since HER2 mutations are heterogeneous, it is difficult to develop drugs [103, 104]. Since they are located next to the flexible motif, HER2 insertion mutations cause a decrease in the helix αC flexibility by stabilizing the loop region [98]. In‐frame non‐frameshift exon 20 insertion mutations (exon20ins) are the most frequently seen type of HER2 mutations (Figure 7b), corresponding to the nine‐codon region in exon 20, including insertions and duplications, which results in aberrant kinase activity compared with HER2^WT^ due to the narrowing of the ATP‐binding cleft [25], and the primary variant is the A775_G776insYVMA mutation (YVMAins) which is led by recurrent 12 base‐pair insertion leading to the duplication of the four amino acid residues YVMA [105]. HER2 exon20ins are responsible for constitutive activation by affecting AKT/MEK pathways [106], and according to the molecular dynamics (MD) simulations, these mutations lead to ligand‐independent activation by restricting HER2 to its active state [104]. In addition, HER2^YVMA^ provides EGFR activation in the absence of EGFR kinase activity or ligands [25], which is why especially this variant shows resistance to pan‐HER‐TKIs [107, 108, 109]. This is mainly due to the alterations in the spatial conformation of the αC helix and the phosphate‐binding loop caused by YVMAins, which allow both to move into the drug‐BP of the HER2‐TKD. This results in compression, narrowing, and steric hindrance of the drug‐BP, in that way hindering the effective binding of most TKIs [110].

### 2.5 EGFR and HER2 Tyrosine Kinase Inhibitors for Cancer Treatment

**Figure 8:**
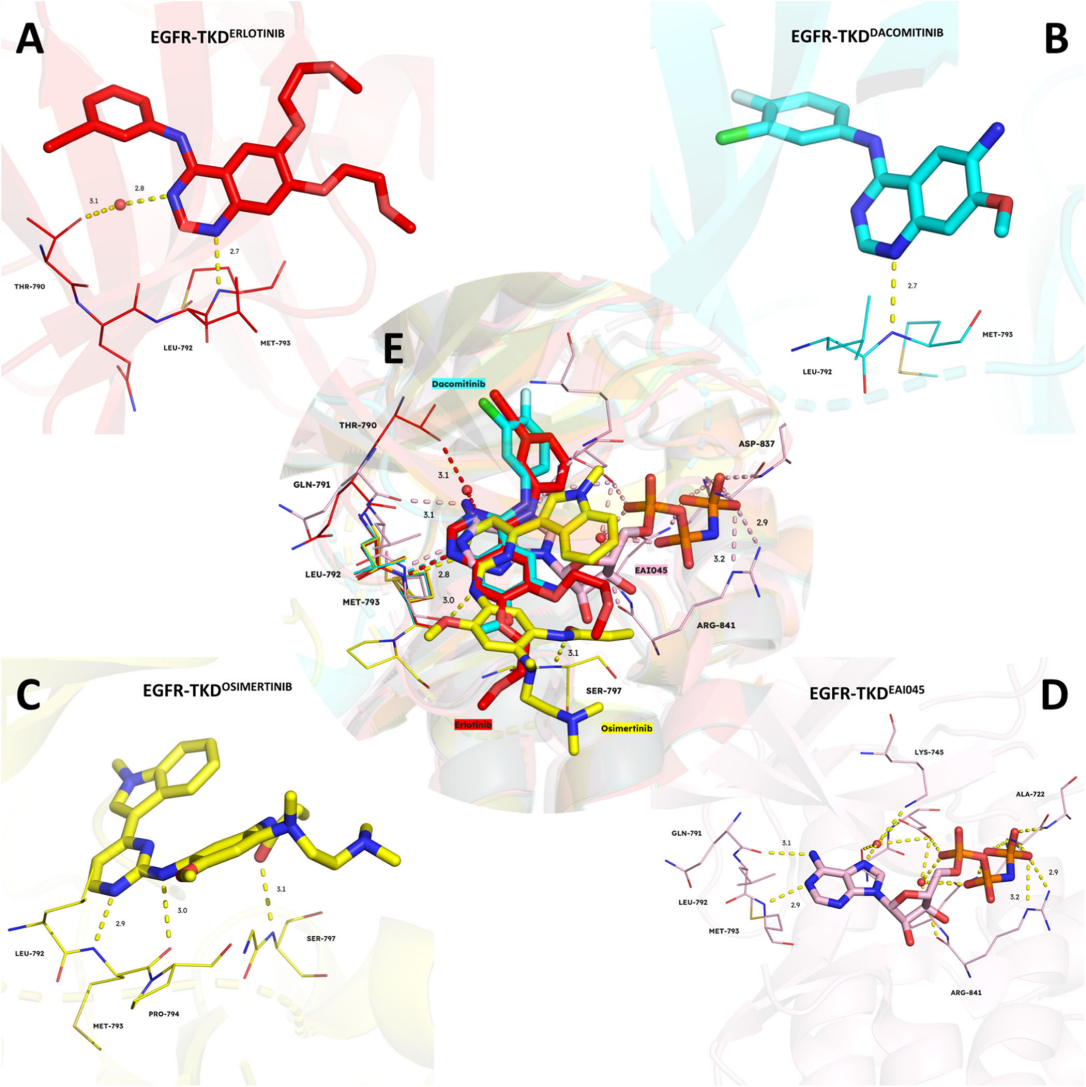
Binding of the EGFR tyrosine kinase inhibitors. (A) EGFR‐TKD bound with first‐generation TKI erlotinib (red, PDB: 1M17), (B) second‐generation TKI dacomitinib (cyan, PDB: 4I23), (C) third‐generation TKI osimertinib (yellow, PDB: 6LUD), (D) fourth‐generation TKI EAI045 (pink, PDB: 6P1L), and E) the alignment of four generations of EGFR‐TKIs. As it can be observed, each generation of inhibitors binds to different regions of TKD. Figure created using PyMOL (PyMOL Molecular Graphics System, Version 3.0, Schrödinger LLC). (See also the Supporting Information).

**Figure 9:**
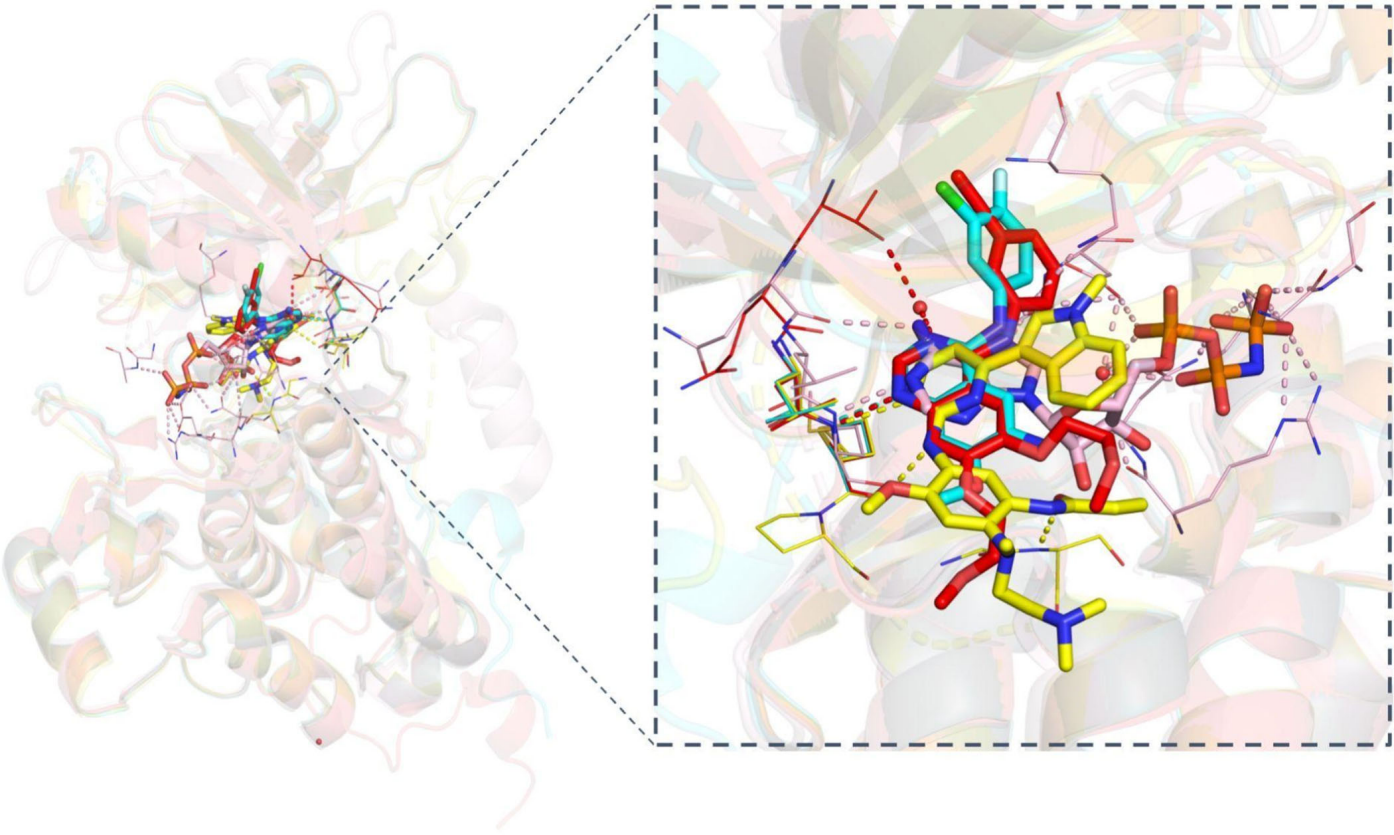
A global view of EGFR‐TKD with inhibitors. The binding sites of the first‐generation, second‐generation, third‐generation, and fourth‐generation EGFR tyrosine kinase inhibitors. Figure created using PyMOL (PyMOL Molecular Graphics System, Version 3.0, Schrödinger LLC). (See also the Supporting Information).

EGFR and HER2‐TKIs have become a standard treatment for NSCLC patients with EGFR mutations. These inhibitors work by inhibiting kinase activity, ultimately blocking downstream signaling. Nevertheless, multiple generations of EGFR and HER2‐TKIs had to be developed to overcome different mutations that have occurred and gave rise to resistance to anterior generations. These inhibitors bind to different regions of TKD and act via different molecular mechanisms (Figures 8 and 9).

The first‐generation EGFR‐TKI gefitinib obtained the approval of the U.S. Food and Drug Administration (FDA) in 2003 for treatments of patients with NSCLC and became the first EGFR‐TK inhibitor to be clinically approved [111]. Another first‐generation EGFR inhibitor, erlotinib, followed gefitinib and received approval from the FDA in 2004 [112]. These first‐generation inhibitors bind reversibly and competitively to the ATP BP of the intracellular tyrosine kinase domain and, therefore, inhibit autophosphorylation events [113]. However, their reversible binding nature renders these first‐generation inhibitors ineffective against resistance mechanisms [114]. It was also understood that gefitinib was confined to patients with exon 19 deletion and exon 21 L858R mutations, which make EGFR sensitive to first‐generation inhibitors [115].

To overcome this resistance problem, second‐generation irreversible inhibitors such as afatinib, dacomitinib, and neratinib were developed [116, 117, 118]. These second‐generation inhibitors contain quinazoline moiety similar to that of gefitinib. However, their side chains can bind irreversibly to C797 in the TKD via Michael addition to the acrylamide group of TKIs and, therefore, inhibit autophosphorylation events by covalently alkylating C797 residue [119, 120]. Unfortunately, most of these second‐generation inhibitors cause severe side effects and face dose‐limiting toxicity and very low maximum‐tolerated doses, which highly limit their usage in the treatments [121].

Resistance to first‐ and second‐generation inhibitors led to the development of mutant selective third‐generation inhibitors for treating NSCLC patients [120]. Most of the third‐generation inhibitors contain aminopyrimidine moiety that covalently binds to active thiols of C797; therefore, they target T790M mutants selectively and irreversibly [122]. However, multiple mechanisms have been identified to cause resistance against third‐generation EGFR inhibitors; in fact, the most common one is the C797S mutation [123, 124]. The tertiary mutation C797S prevents the covalent bond formation between inhibitors and the C797 at the ATP BP due to the substitution of less reactive serine, which cannot act as a Michael donor to the acrylamide group of the inhibitors in place of cysteine at position 797 within exon 20 [125], therefore, it results in acquired resistance.

Recently, scientists have been trying to develop newer alternatives, the fourth‐generation EGFR‐TK inhibitors, to combat C797S resistance mutation. The fourth‐generation inhibitors are allosteric kinase inhibitors, which offer promising results over competitive inhibitors due to their specific binding to the target and selectively inhibiting the mutants [126].

### 2.6 Beyond Our Perspective: Pyrazoline‐Thiazole Hybrids as Promising EGFR and HER2 Inhibitors

**Figure 10:**
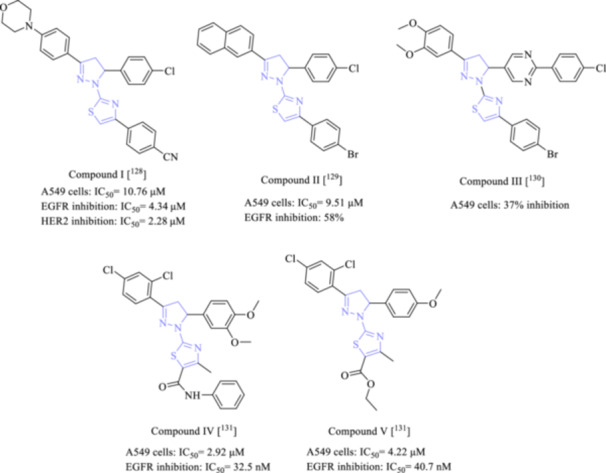
Pyrazoline‐thiazole scaffolded EGFR and/or HER2 inhibitors as potential anti‐NSCLC agents.

To date, a wide range of small molecules, both synthetic and natural, have been identified that can inhibit EGFR [127]. One of the main themes of our research group is the discovery of new compounds effective against NSCLC through EGFR and HER2 inhibition. We previously synthesized pyrazoline‐thiazole‐containing compounds and investigated their anticancer effects against NSCLC through EGFR and HER2 inhibition [128, 129, 130]. The hybridization of pyrazoline–thiazole scaffolds has long been known to be effective against NSCLC as EGFR and/or HER2 inhibitors [128, 129, 130, 131]. Compound I [128] (Figure 10) was detected to show anti‐NSCLC effect with an IC_50_ value of 10.76 μM compared with erlotinib (IC_50_ = 22.35 μM). This compound also significantly inhibited both EGFR and HER2 with IC_50_ values of 4.34 and 2.28 μM, respectively. In our other study, we synthesized pyrazoline–thiazole hybrids and determined that compound II [129] revealed potential anti‐NSCLC activity with an IC_50_ value of 9.51 µM compared with lapatinib (IC_50_ = 16.44 μM). Besides, compound II (Figure 10) selectively inhibited EGFR (58.32%) without inhibiting HER2 at 10 µM concentration. In our most recent study [130], 37% of NSCLC cells were found sensitive against compound III (Figure 10). The starting materials of compound III, the propenone and pyrazoline–carbothioamide derivatives, revealed more significant anticancer effects against NSCLC cells compared with compound III. We are continuing to pursue pyrazoline‐thiazole‐based drug candidates for the treatment of NSCLC.

In other study, Abdelsalam et al. 2022 [131], synthesized thiazolyl–pyrazoline derivatives and showed that two compounds (Compounds IV and V) (Figure 10) in this series were found to be effective against NSCLC cell lines. Compounds IV and V also exhibited notable EGFR inhibition with IC_50_ values of 32.5 and 40.7 nM, respectively.

## 3 Conclusion and Future Remarks

The therapeutic landscape for EGFR‐mutant non‐small‐cell lung cancer (NSCLC) is evolving, with third‐generation EGFR tyrosine kinase inhibitors (TKIs) as the first‐line standard. However, resistance remains a major challenge, driving the need for novel therapeutic combinations and emerging strategies [132, 133]. Fourth‐generation EGFR TKIs, such as EAI045, BDTX‐1535, and TAS3351, offer promise against resistance mutations like EGFR^C797S^ and are under clinical investigation [134]. These agents are designed to enhance potency, selectivity, and central nervous system penetration. The fourth‐generation TKIs are expected to address resistance mechanisms such as triple mutations, including EGFR^L858R/T790M/C797S^, more effectively than their predecessors [132].

Combining third‐generation EGFR TKIs with antiangiogenic agents [132, 135], chemotherapy [132, 136], or antibody‐drug conjugates (ADCs), including EGFR‐MET bispecific antibodies like amivantamab [132, 137, 138] has also shown promise in delaying resistance. Targeted protein degraders are being developed to selectively target resistant cancer cells [133, 139], while circulating tumor DNA (ctDNA) analysis is emerging as a tool for guiding treatment adjustments, enabling a shift toward more personalized therapy [132, 140, 141]. These advances, along with emerging therapeutic approaches, pave the way for more adaptive and personalized treatment strategies that integrate molecular diagnostics with multi‐modal interventions. Ongoing clinical trials will be instrumental in determining the optimal combination of these novel agents to maximize patient outcomes. The drug design strategy should be focused on developing therapies that overcome acquired resistances and refining TKI treatments for specific NSCLC biomarkers to ensure more effective and targeted treatments.

Our review underscores the critical roles of the ErbB receptors, particularly EGFR and HER2, in the pathogenesis of non‐small cell lung cancer. The structural insights into these receptors highlight their complex conformational dynamics, which are fundamental to their function and relevance in cancer therapy. We have observed significant progress in understanding the active conformations of the EGFR tyrosine kinase domain, yet the characterization of the HER2 tyrosine kinase domain remains incomplete, particularly regarding its active and inactive states. Furthermore, the absence of crystal structures for the EGFR‐HER2 heterodimer tyrosine kinase domains represents a significant gap that impedes our understanding of their combined role in NSCLC.

Future structural studies should prioritize obtaining high‐resolution structures of HER2‐TKD and the EGFR‐HER2 heterodimer to fully elucidate their activation mechanisms and resistance profiles. Detailed structural studies are essential for revealing the complete range of conformational states and interactions that influence drug binding and efficacy. Such advancements will enhance structure‐based drug design, leading to more effective treatments and ultimately improving survival and quality of life for patients with NSCLC.

By advancing our understanding of EGFR and HER2 structures, we pave the way for innovative therapies. Addressing the current limitations and resistance issues will be key to developing effective treatments and achieving better clinical outcomes for NSCLC patients.

## Supporting information

Supporting information.

## Data Availability

The data that support the findings of this study are available from the corresponding author upon reasonable request.
