# Supplementary material for: A Structural Insight Into Two Important ErbB Receptors (EGFR and HER2) and Their Relevance to Non‐Small Cell Lung Cancer
Source: Arch Pharm (Weinheim). 2025 Apr 7;358(4):e2400992. doi: 10.1002/ardp.202400992 (PMC11975551; doi:10.1002/ardp.202400992)
Supplement: Supplementary file 1 — Supporting information. [file ARDP-358-e2400992-s001.docx]

**Supporting Information**

**A Structural Insight into Two Important ErbB Receptors (EGFR and HER2) and Their Relevance to Non-Small Cell Lung Cancer**

*Edanur Topalan^1^, Ahmet Büyükgüngör^1,2^, Melih Çiğdem^1,3^, Sinan Güra^1,4^, Belgin Sever^5,6^, Masami Otsuka^6,7^, Mikako Fujita^6^, Hasan Demirci^1,*^, Halilibrahim Ciftci^6,7,8,9,*^*

*^1^Department of Molecular Biology and Genetics, Koc University, 34450, Istanbul, Türkiye; etopalan23@ku.edu.tr; hdemirci@ku.edu.tr*

*^2^Department of Molecular Biology and Genetics, Istanbul Technical University, 34469, Istanbul, Türkiye; buyukgungor21@itu.edu.tr*

*^3^Department of Biological Sciences, Middle East Technical University, 06800, Ankara, Türkiye; melih.cigdem@metu.edu.tr*

*^4^Graduate School of Biology & Health, Université Paris Saclay, 91400, Orsay, France; sinan.gura@universite-paris-saclay.fr*

*^5^ Department of Pharmaceutical Chemistry, Faculty of Pharmacy, Anadolu University, Eskisehir 26470, Türkiye;* *belginsever@anadolu.edu.tr*

*^6^ Medicinal and Biological Chemistry Science Farm Joint Research Laboratory, Faculty of Life Sciences, Kumamoto University, Kumamoto 862-0973, Japan; motsuka@gpo.kumamoto-u.ac.jp; mfujita@kumamoto-u.ac.jp*

*^7^ Department of Drug Discovery, Science Farm Ltd., Kumamoto 862-0976, Japan*

*^8^Department of Molecular Biology and Genetics, Mehmet Akif Ersoy University, Burdur 15030, Türkiye; hciftci@mehmetakif.edu.tr*

*^9^Department of Bioengineering Sciences, Izmir Katip Celebi University, Izmir 35620, Türkiye*


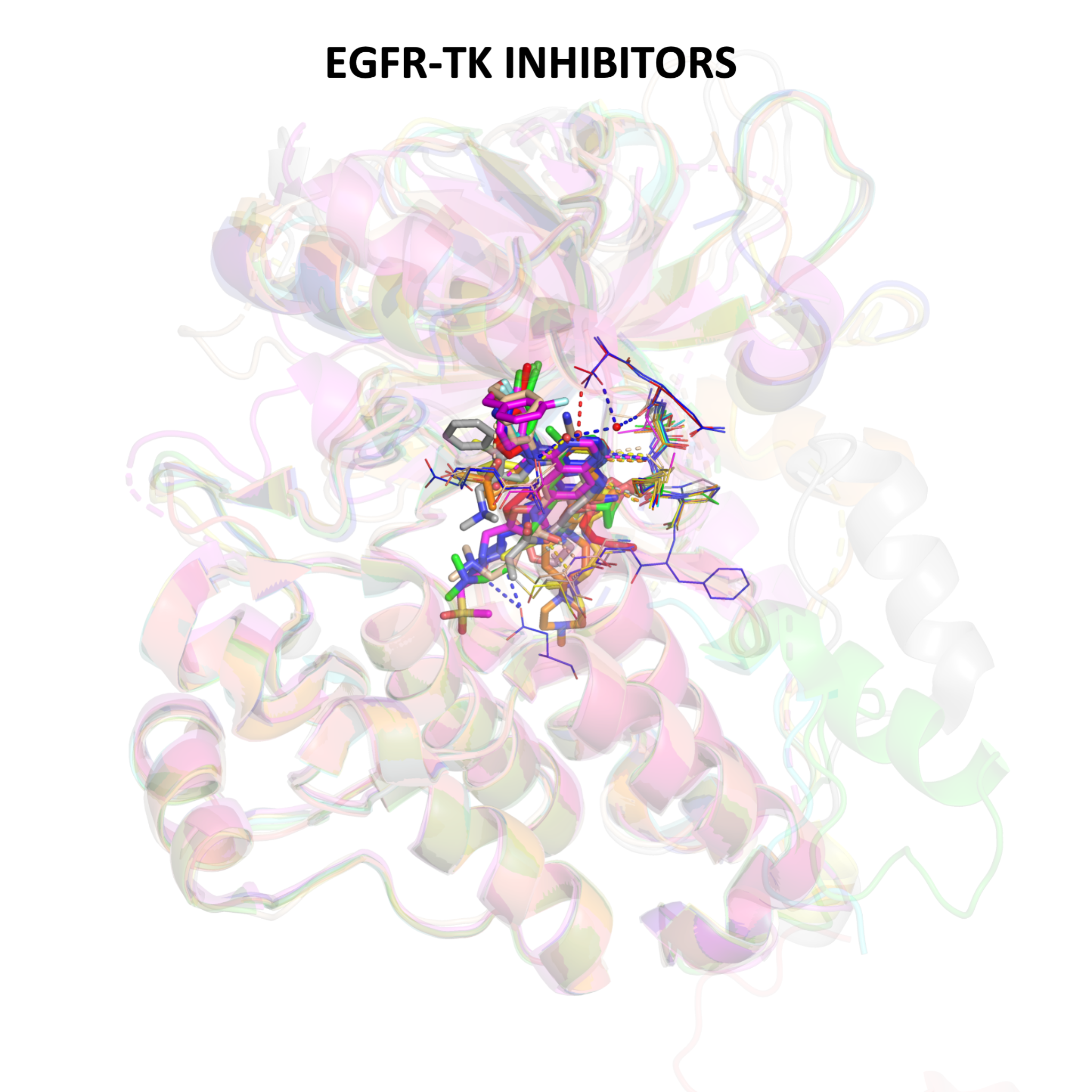


**Supplementary Figure 1.** A global view of EGFR-TKD and the binding sites of the EGFR tyrosine kinase inhibitors.


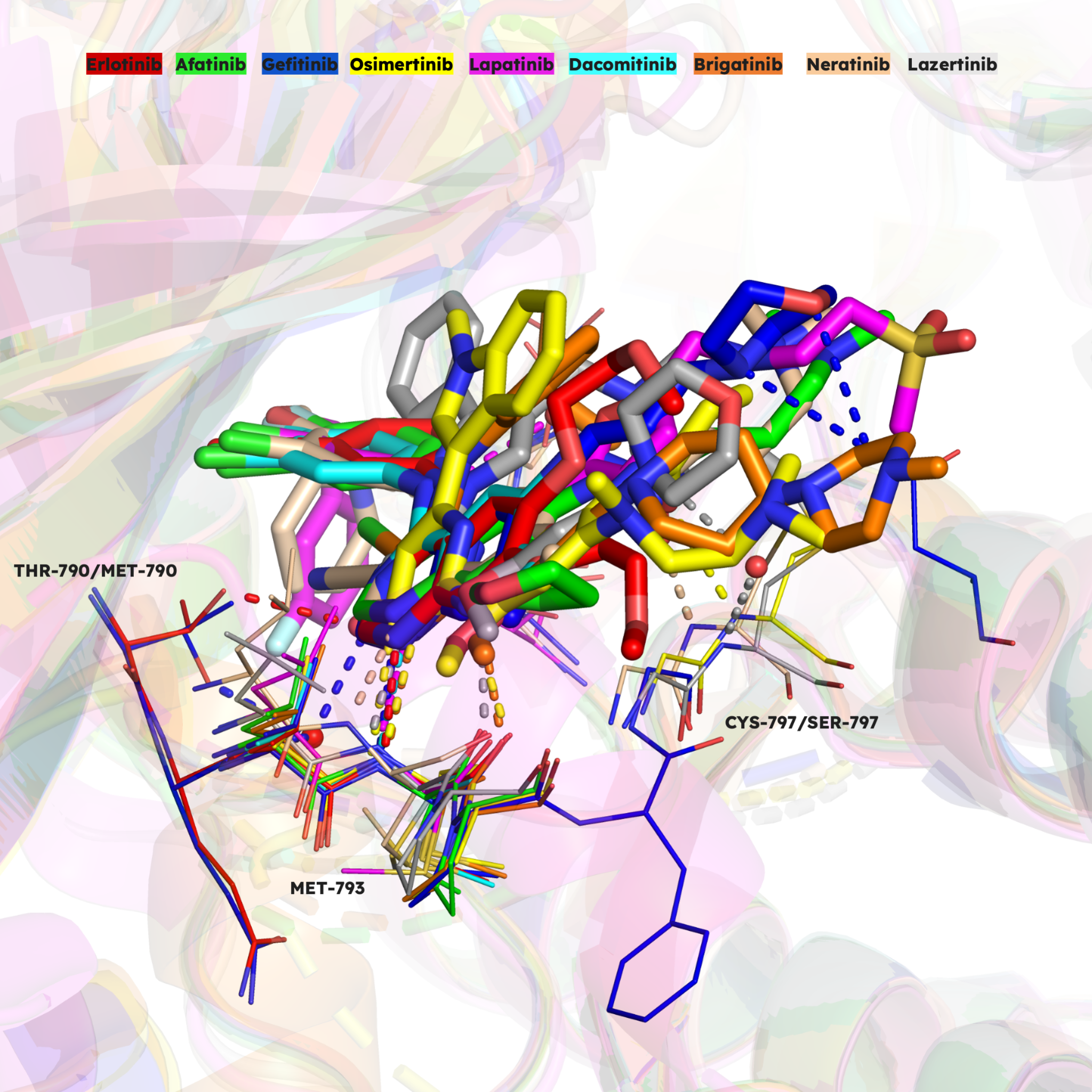


**Supplementary Figure 2.** A zoom-in to the EGFR-TKD bound with TKI drugs erlotinib (red, PDB: 1M17), afatinib (green, PDB: 4G5J), gefitinib (blue, PDB: 4WKQ), osimertinib (yellow, PDB: 6LUD), lapatinib (pink, PDB: 1XKK), dacomitinib (cyan, PDB: 4I23), brigatinib (orange, 7ZYM), and neratinib (wheat, PDB: 2JIV).


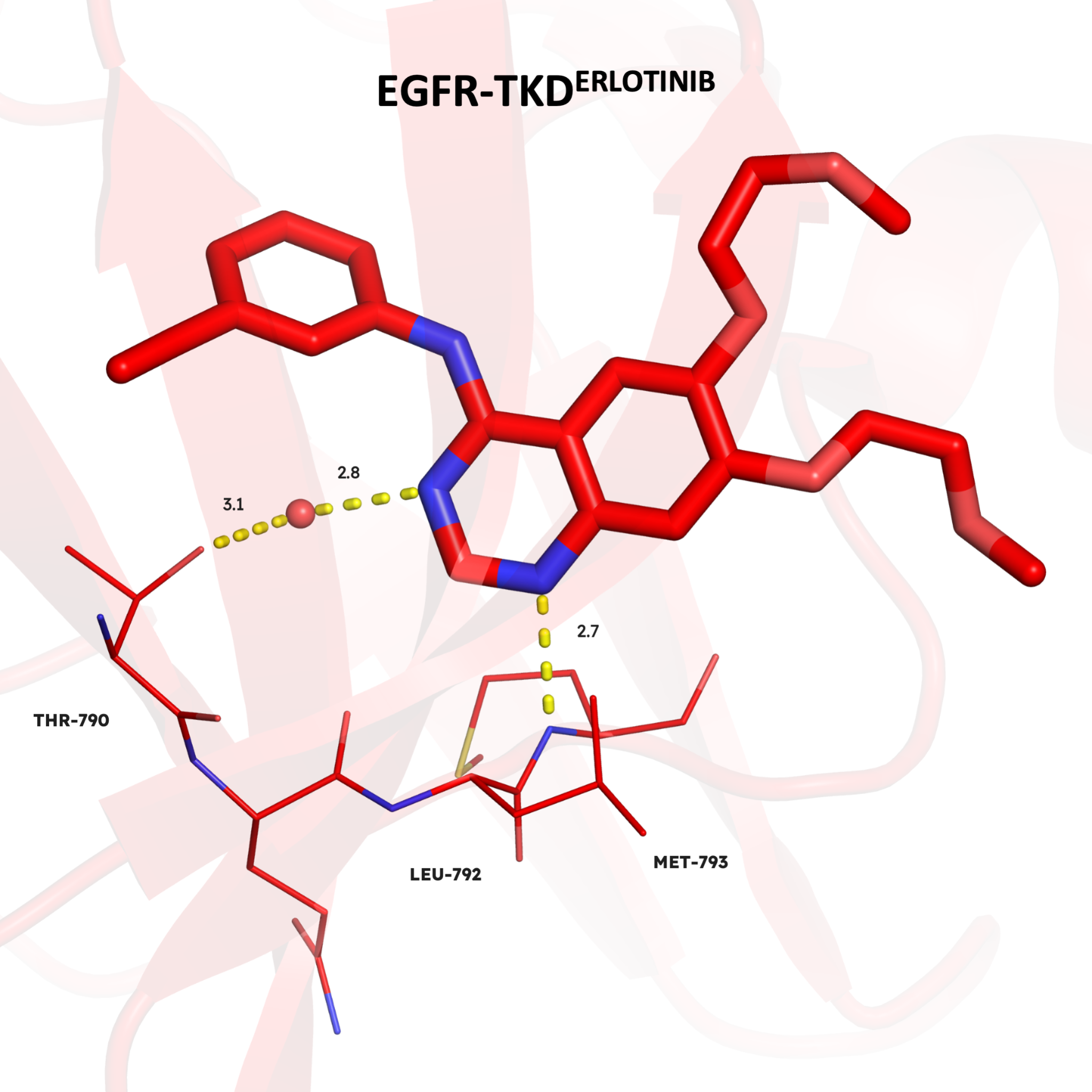


**Supplementary Figure 3.** EGFR-TKD bound with TKI drugs erlotinib (red, PDB: 1M17).

**
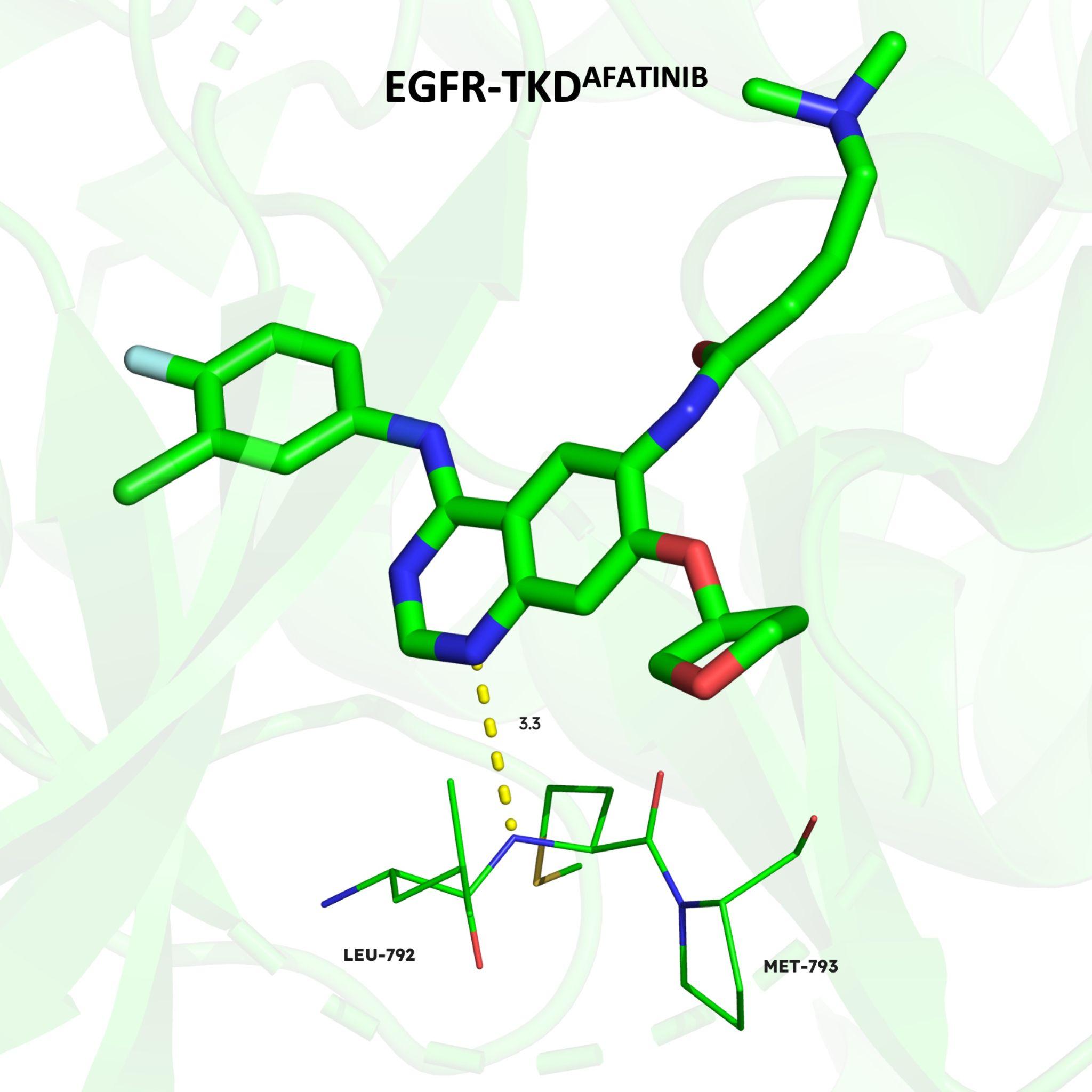
**

**Supplementary Figure 4.** EGFR-TKD bound afatinib (green, PDB: 4G5J).


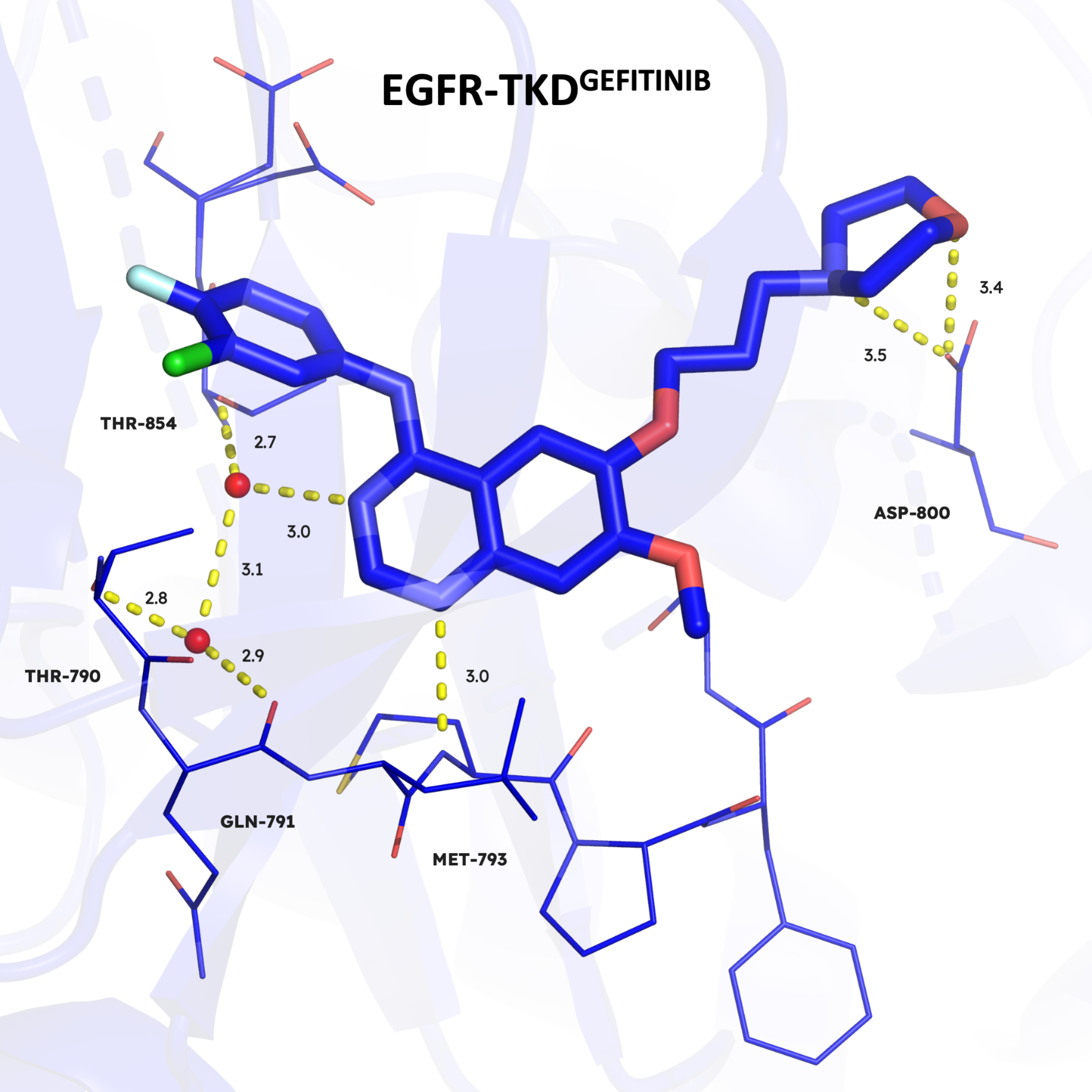


**Supplementary Figure 5.** EGFR-TKD bound with gefitinib (blue, PDB: 4WKQ).

**
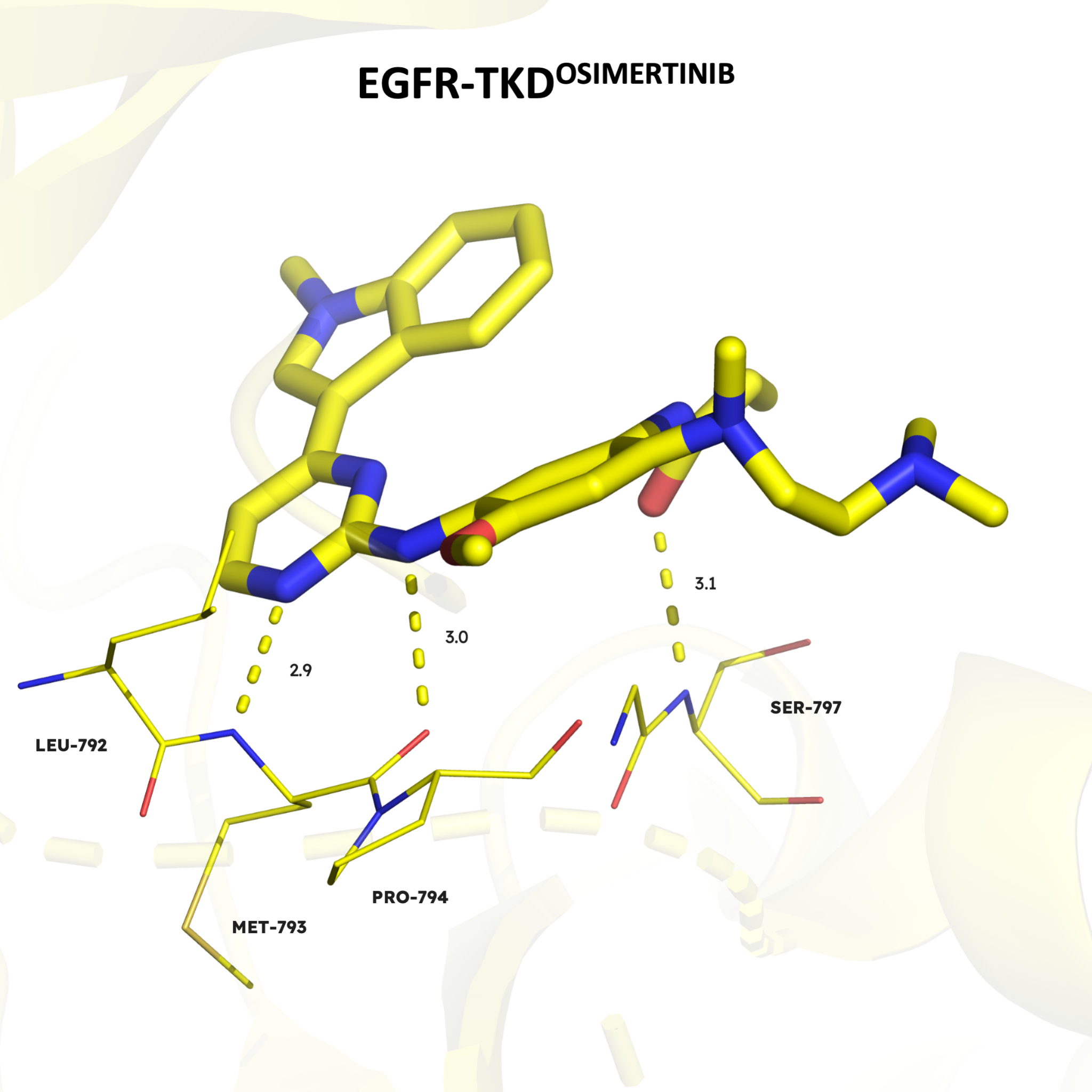
**

**Supplementary Figure 6.** EGFR-TKD bound with osimertinib (yellow, PDB: 6LUD).


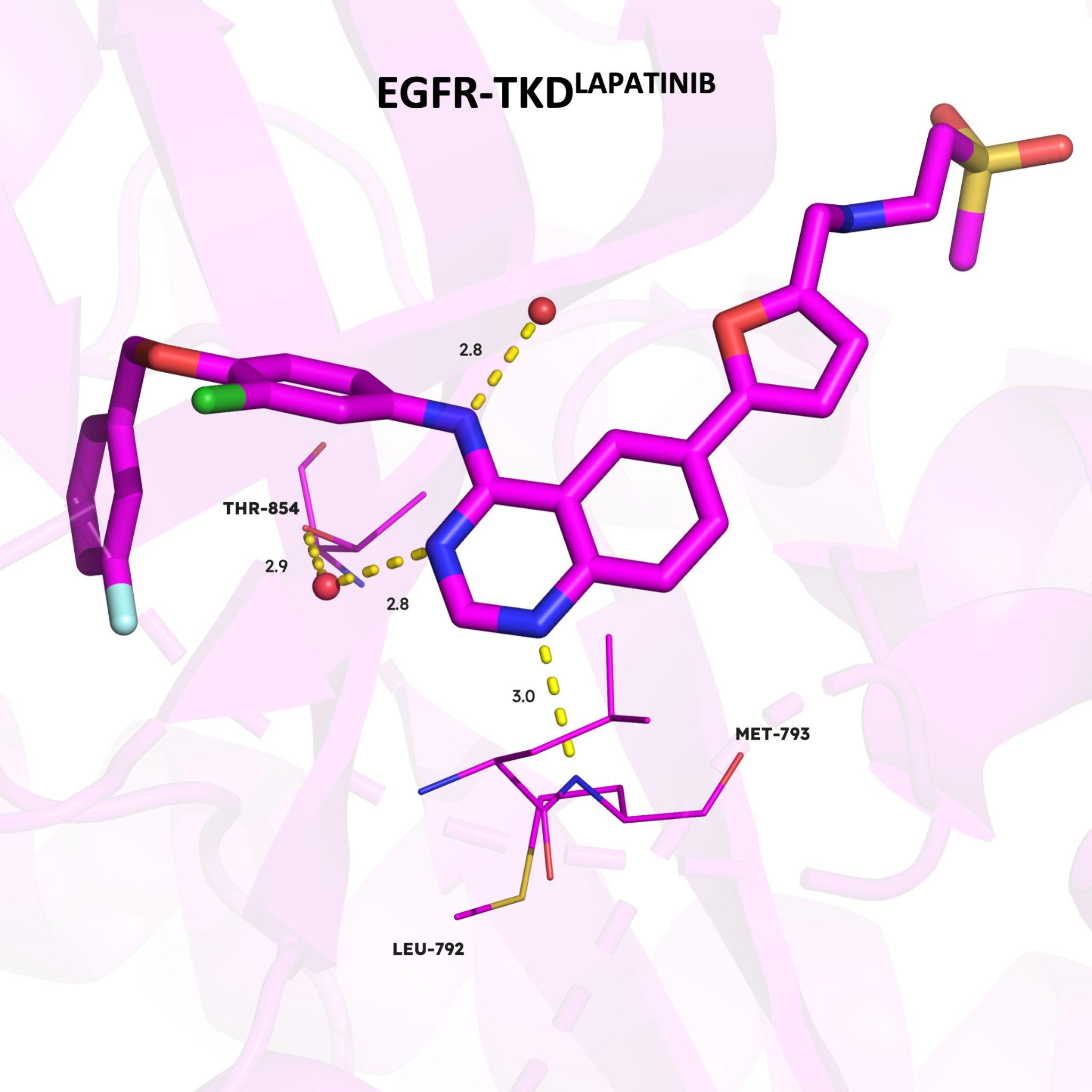


**Supplementary Figure 7.** EGFR-TKD bound with lapatinib (pink, PDB: 1XKK).


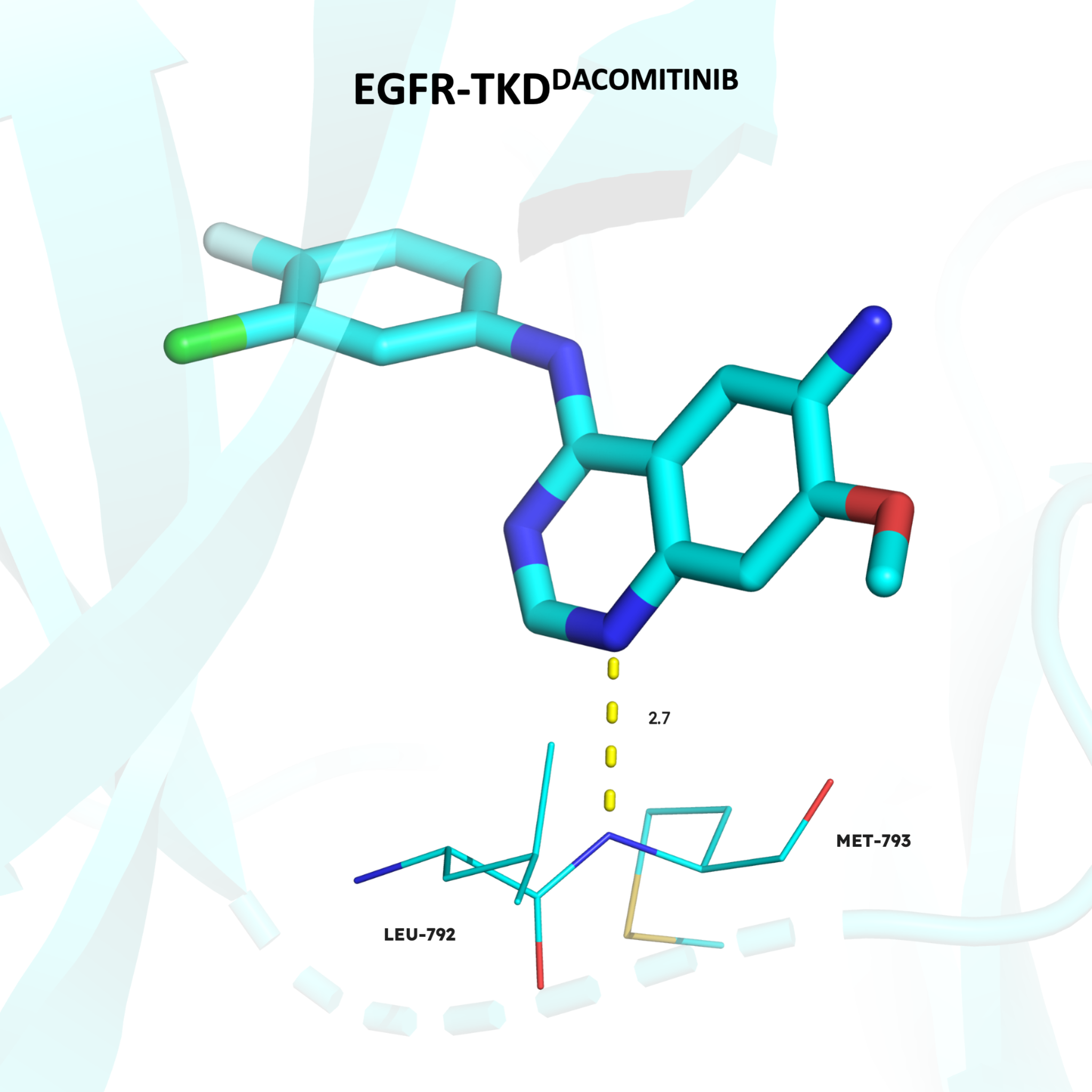


**Supplementary Figure 8.** EGFR-TKD bound with dacomitinib (cyan, PDB: 4I23).


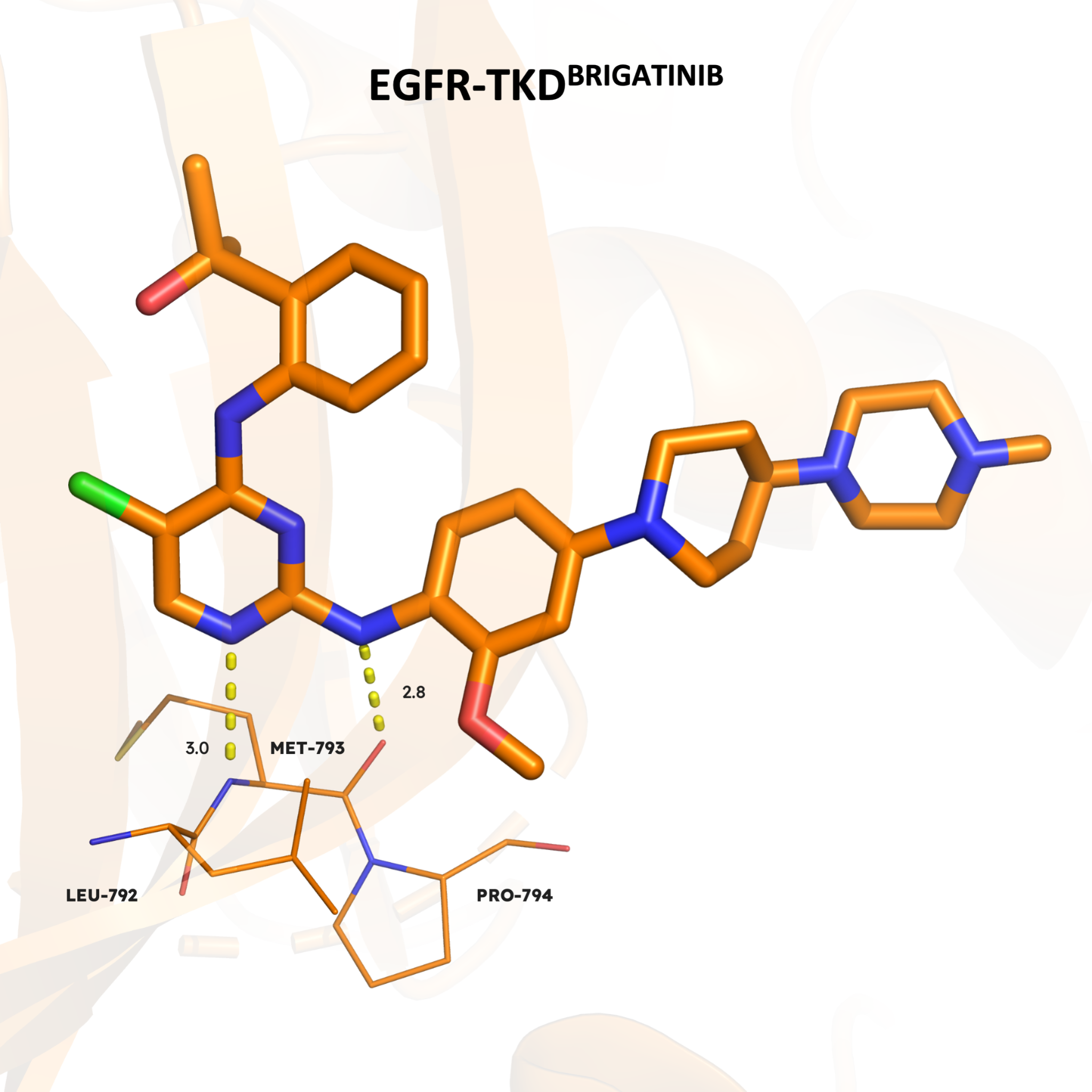


**Supplementary Figure 9.** EGFR-TKD bound with brigatinib (orange, 7ZYM).


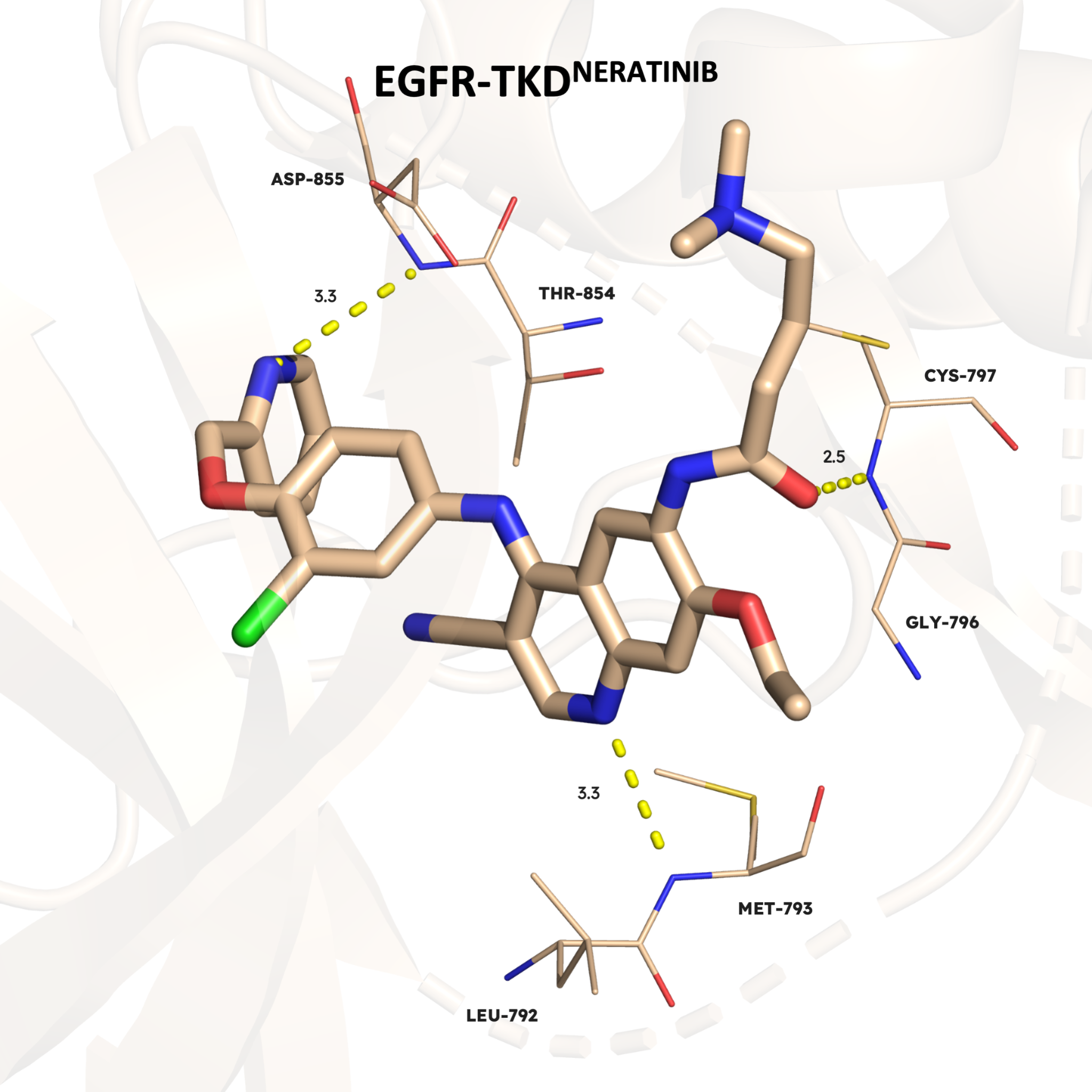


**Supplementary Figure 10.** EGFR-TKD bound with neratinib (wheat, PDB: 2JIV).


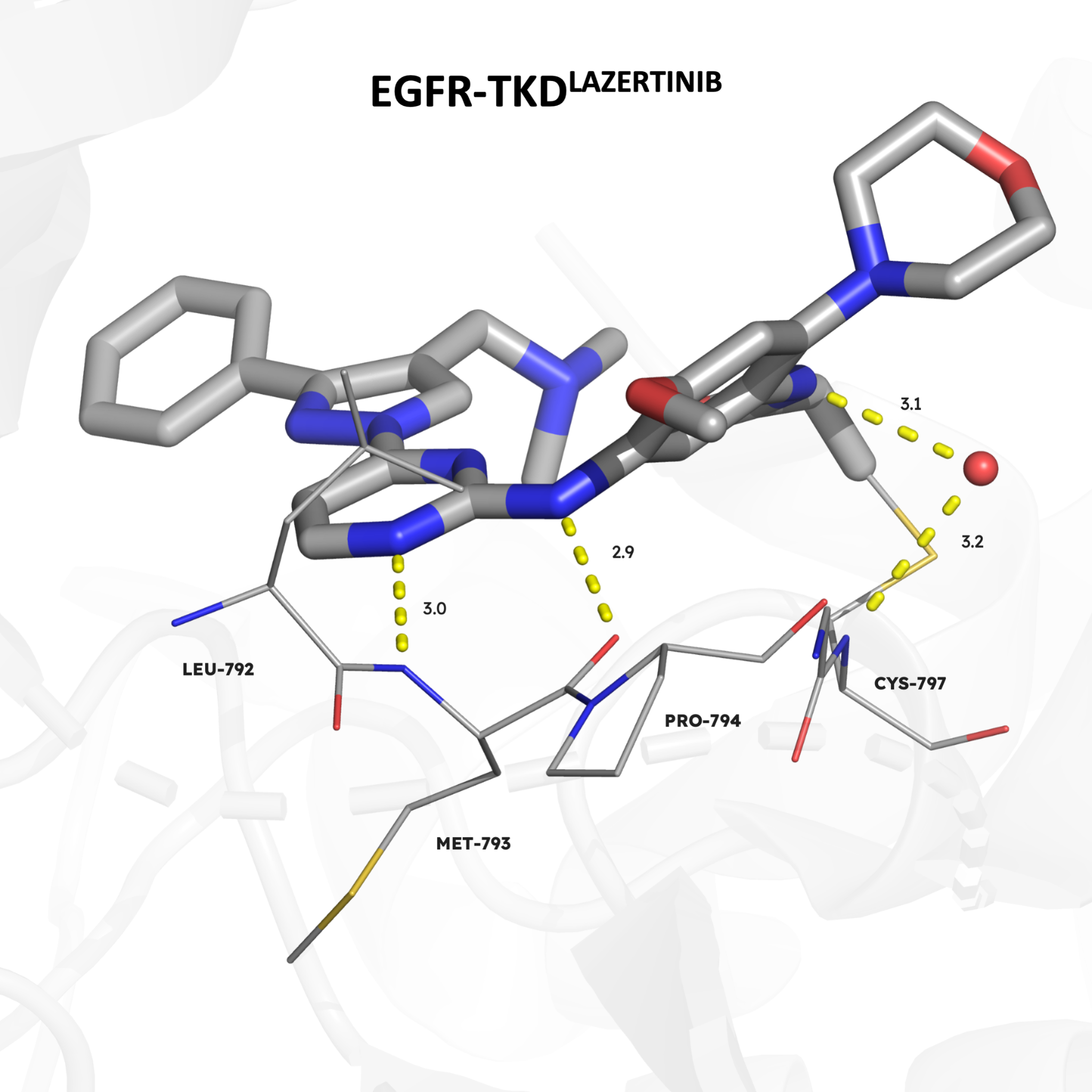


**Supplementary Figure 11.** EGFR-TKD bound with lazertinib (white, PDB: 7UKW).


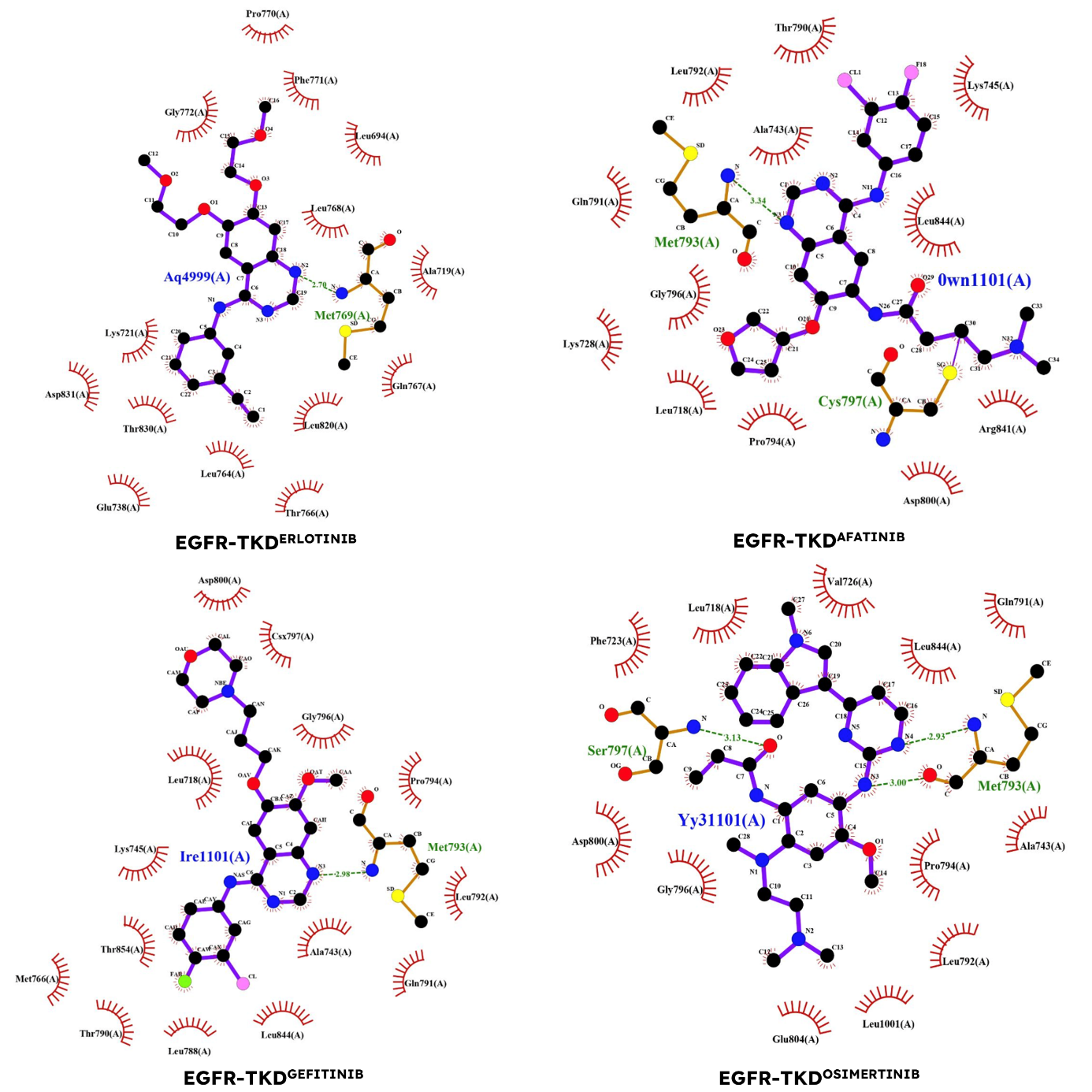


**Supplementary Figure 12.** EGFR-TKD^ERLOTINIB^ (PDB 1M17), EGFR-TKD^AFATINIB^ (PDB 4G5J), AGFR-TKD^GEFITINIB^ (PDB 4WKQ), EGFR-TKD^OSIMERTINIB^ (PDB 6LUD).


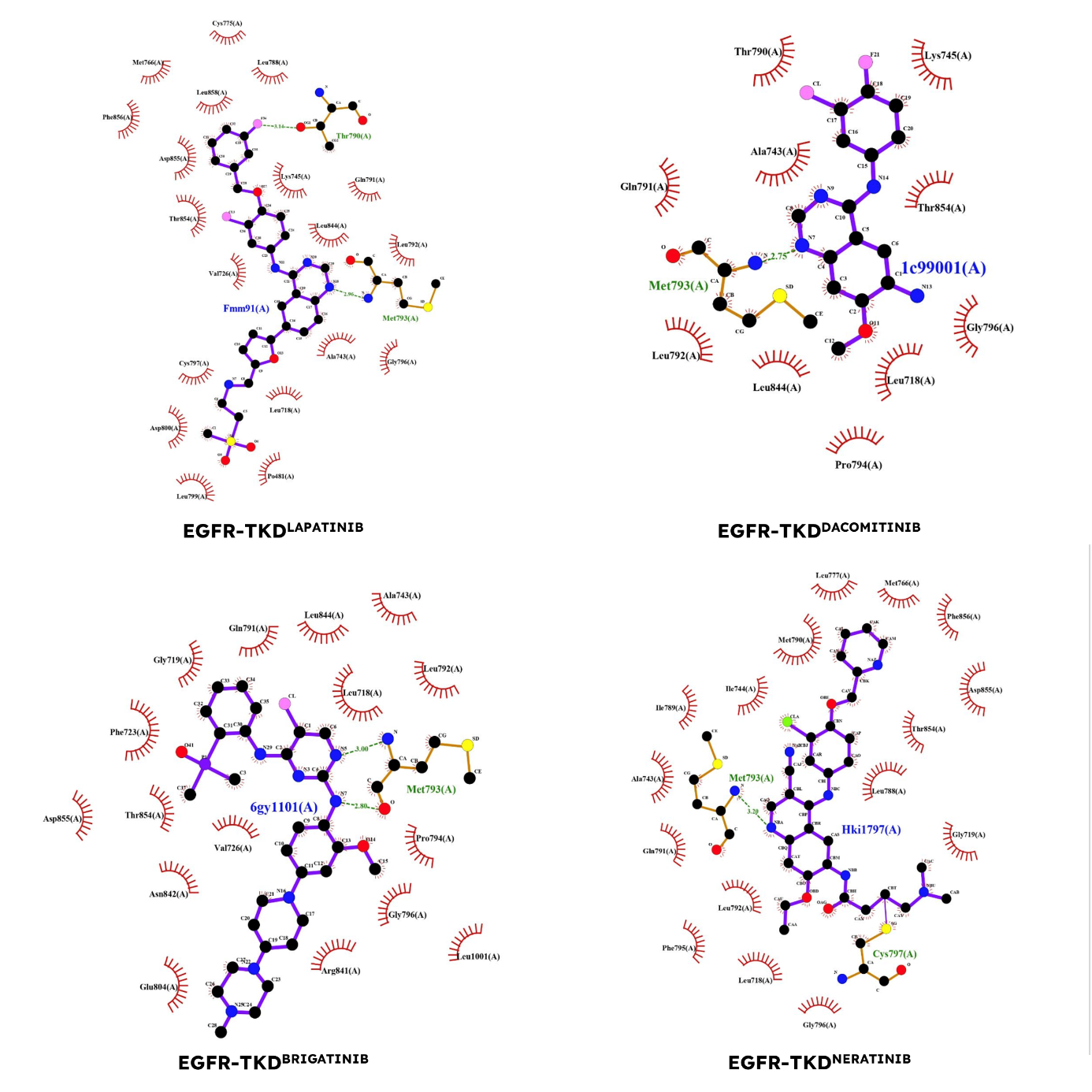


**Supplementary Figure 13.** EGFR-TKD^LAPATINIB^ (PDB 1XKK), EGFR-TKD^DACOTIMINIB^ (PDB 4I23), EGFR-TKD^BRIGATINIB^ (PDB 7ZYM), EGFR-TKD^NERATINIB^ (PDB 2JIV).


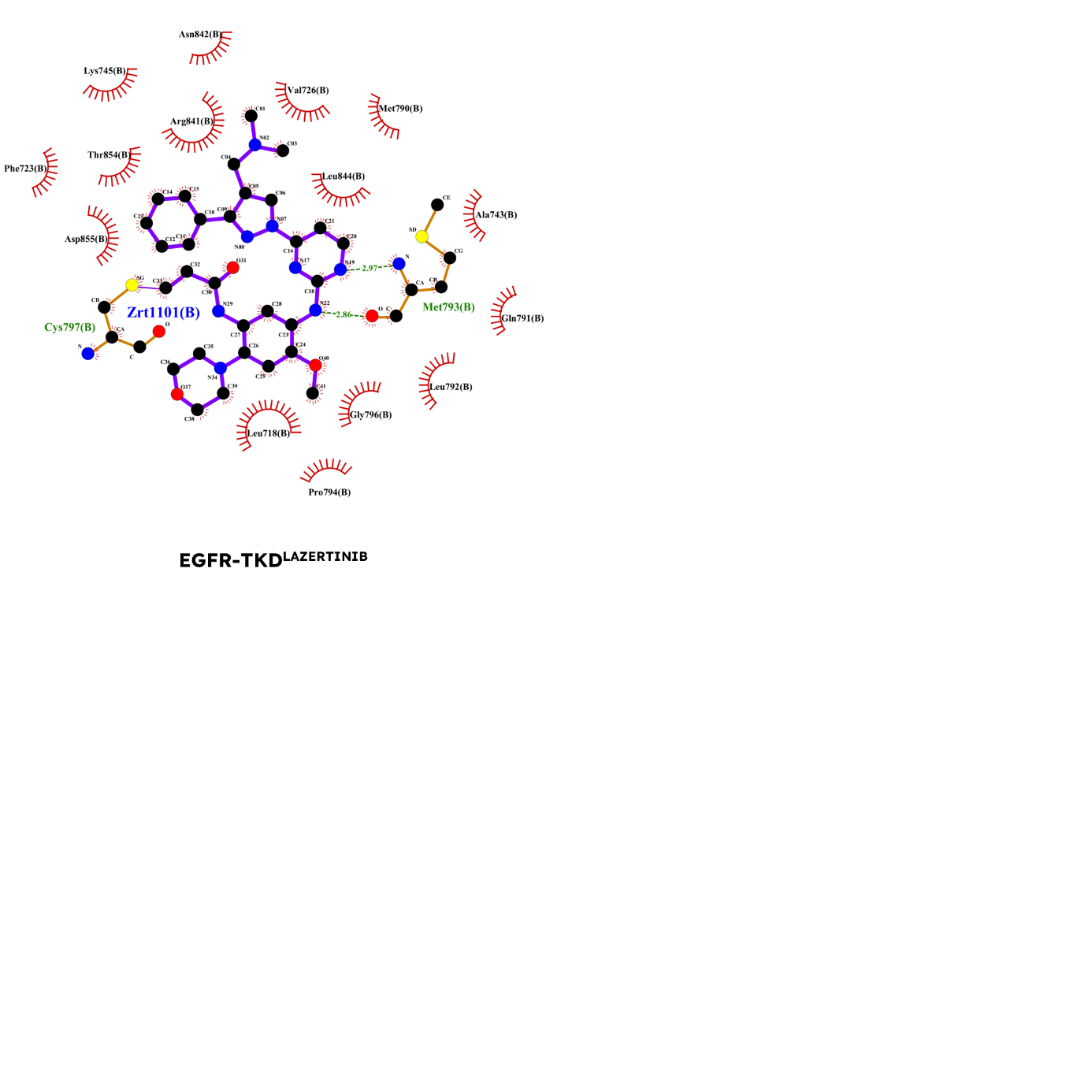


**Supplementary Figure 14.** EGFR-TKD^LAZERTINIB^ (PDB 7UKW).

**Supplementary Table 1.** FDA-approved small-molecule tyrosine kinase domain inhibitors (TKI) of EGFR. Three generations of TKIs, as well as their molecular target of inhibition are illustrated in the table, each one with a different color code (1st generation: green; 2nd generation: blue; 3th generation: yellow). The 4th generation of EGFR-TKIs are currently undergoing preclinical evaluation and therefore are not present in the table.

| **Inhibitor Name** | **Generation G**  **Mode of Action** | **Molecular Target** | **Tumor Type** | **Year of Approval** |
| --- | --- | --- | --- | --- |
| Gefitinib | 1st Reversible,  Competitive | ATP binding site of EGFR TKD | Advanced or metastatic NSCLC, pancreatic cancer | 2003 |
| Erlotinib | 1st Reversible, Competitive | ATP binding site of EGFR TKD | Locally advanced or metastatic NSCLC, pancreatic cancer | 2004 |
| Lapatinib | 1st Reversible,  Competitive | ATP binding site of EGFR C HER2 TKD | Metastatic breast cancer, gastric cancer | 2007 |
| Icotinib | 1st  Reversible, Competitive | ATP binding site of EGFR TKD | Advanced or metastatic NSCLC | 2011 |
| Afatinib | 2nd Covalently binding,  irreversible | ATP binding site of EGFR, HER2, and HER4 | Metastatic NSCLC, breast cancer | 2013 |
| Neratinib | 2nd Covalently binding,  irreversible | ATP binding site of EGFR, HER2,  and HER4 | HER2-positive breast cancer | 2017 |
| Brigatinib | 2nd Covalently binding,  irreversible | ATP binding site of EGFR TKD | NSCLC, anaplastic large cell lymphoma,  neuroblastomas | 2017 |
| Dacomitinib | 2nd Covalently binding,  irreversible | ATP binding site of EGFR, HER2, and  HER4 | Metastatic NSCLC | 2018 |
| Osimertinib | 3th Covalently binding,  irreversible | ATP binding site of EGFR | Advanced and metastatic NSCLC | 2015 |
| Furmonertinib | 3th Covalently binding,  irreversible | ATP binding site of EGFR | Locally advanced or metastatic NSCLC | 2021 |
| Tucatinib | 3th Covalently binding,  irreversible | ATP binding site of HER2 | HER2-positive breast cancer | 2023 |
| Lazertinib | 3th Covalently binding,  irreversible | ATP binding site of EGFR | Advanced NSCLC | 2024 |
